# Recruitment of PfSET2 by RNA Polymerase II to Variant Antigen Encoding Loci Contributes to Antigenic Variation in *P. falciparum*

**DOI:** 10.1371/journal.ppat.1003854

**Published:** 2014-01-02

**Authors:** Uchechi E. Ukaegbu, Sandeep P. Kishore, Dacia L. Kwiatkowski, Chethan Pandarinath, Noa Dahan-Pasternak, Ron Dzikowski, Kirk W. Deitsch

**Affiliations:** 1 Department of Microbiology and Immunology, Weill Medical College of Cornell University, New York, New York, United States of America; 2 Program in Computational Biology, Weill Medical College of Cornell University, New York, New York, United States of America; 3 Department of Microbiology and Molecular Genetics, The Institute for Medical Research Israel-Canada, The Kuvin Center for the Study of Infectious and Tropical Diseases, Hebrew University-Hadassah Medical School, Jerusalem, Israel; Indiana University School of Medicine, United States of America

## Abstract

Histone modifications are important regulators of gene expression in all eukaryotes. In *Plasmodium falciparum*, these epigenetic marks regulate expression of genes involved in several aspects of host-parasite interactions, including antigenic variation. While the identities and genomic positions of many histone modifications have now been cataloged, how they are targeted to defined genomic regions remains poorly understood. For example, how variant antigen encoding loci (*var*) are targeted for deposition of unique histone marks is a mystery that continues to perplex the field. Here we describe the recruitment of an ortholog of the histone modifier SET2 to *var* genes through direct interactions with the C-terminal domain (CTD) of RNA polymerase II. In higher eukaryotes, SET2 is a histone methyltransferase recruited by RNA pol II during mRNA transcription; however, the ortholog in *P. falciparum* (PfSET2) has an atypical architecture and its role in regulating transcription is unknown. Here we show that PfSET2 binds to the unphosphorylated form of the CTD, a property inconsistent with its recruitment during mRNA synthesis. Further, we show that H3K36me3, the epigenetic mark deposited by PfSET2, is enriched at both active and silent *var* gene loci, providing additional evidence that its recruitment is not associated with mRNA production. Over-expression of a dominant negative form of PfSET2 designed to disrupt binding to RNA pol II induced rapid *var* gene expression switching, confirming both the importance of PfSET2 in *var* gene regulation and a role for RNA pol II in its recruitment. RNA pol II is known to transcribe non-coding RNAs from both active and silent *var* genes, providing a possible mechanism by which it could recruit PfSET2 to *var* loci. This work unifies previous reports of histone modifications, the production of ncRNAs, and the promoter activity of *var* introns into a mechanism that contributes to antigenic variation by malaria parasites.

## Author Summary

Chemical modifications to histones, the proteins that serve as the primary units of chromatin, often determine whether specific genes are actively transcribed or condensed into transcriptionally silent regions of the genome. In the malaria parasite *Plasmodium falciparum*, histone modifications have been shown to play a significant role in controlling gene expression involved in many aspects of their lifecycle, including the complex gene expression patterns associated with antigenic variation. The various histone modifications that are found within the parasite's genome have now been extensively cataloged, and the enzymes that are responsible for adding and removing them have been identified. However, how these enzymes are recruited to specific regions of the genome to coordinate gene expression is not understood. In this paper, we provide the first evidence for recruitment of a unique histone methyltransferase to specific regions of the genome through its tethering to RNA polymerase II. We find that disruption of this interaction results in major changes in expression patterns of genes involved in antigenic variation, demonstrating the importance of regulated recruitment of histone modifiers for parasite biology.

## Introduction

The use of alternative histone modifications is an important mechanism utilized by eukaryotic cells to control many aspects of nuclear biology, including patterns of gene expression, DNA replication, chromosome segregation and overall genome organization. In malaria parasites, histone modifications have been shown to play an important role in regulating many features of host-parasite interactions, including red blood cell invasion [1]–[3], expression of nutrient uptake channels [4], and antigenic variation [5]–[8]. Systematic, genome-wide analysis of the presence and/or absence of various histone modifications have now been conducted, providing a catalog of the epigenetic marks found in different regions of the *Plasmodium falciparum* genome [6], [8]. In addition, analyses of the proteins encoded in the parasite's genome has enabled the identification of many of the enzymes responsible for deposition of these histone modifications [9], and knockouts of select number of these genes have now been conducted [10], [11]. These studies have reinforced the importance of histone modifications in *Plasmodium* biology, and identified these enzymes as potential targets for disease intervention.

While the role histone modifications play in regulating gene expression is well established, an important and poorly understood aspect of this process is how various histone modifiers get directed to specific places within the genome. In some organisms, the production of noncoding RNAs (ncRNAs) has been implicated in the recruitment of various histone modifiers to specific loci [12]. Studies have also shown that once initial marks have been deposited, histone modifications can act combinatorially to enforce or reverse epigenetic marks, thus leading to “cross-talk” between modifications that result in the establishment of different gene expression states [13]. However the mechanism by which the assembly of the proper complement of modifications is initiated is an aspect of chromatin biology that remains in its infancy and virtually no work has been done in *Plasmodium*.

One of the few well-characterized mechanisms of directed recruitment of a histone modifier is that of the SET2 (Su(var)3–9, Enhancer of Zeste, Trithorax 2) class of methyltransferases. These enzymes deposit a tri-methyl group on the lysine in the 36^th^ position of histone H3 (H3K36me3), which can subsequently be removed by its cognate demethylase JmjC1 [14], [15]. In higher eukaryotes, SET2 is recruited by RNA pol II during the act of transcribing mRNA, thereby tethering the protein to the elongating polymerase complex and thus effectively marking regions of the genome that have been transcribed [16], [17]. This characteristic is mediated by the binding of SET2 directly to the C-terminal domain (CTD) of the enzymatic subunit of the polymerase, Rpb1, but only when the CTD has been properly phosphorylated. Phosphorylation of the CTD occurs specifically when the polymerase is engaged in transcriptional elongation [18], [19]. Thus SET2 recruitment coincides directly with mRNA synthesis and deposition of H3K36me3 is limited to specific regions of the genome that have been recently transcribed. This is thought to re-compact chromatin that was loosened by the passage of RNA pol II and thereby prevent inappropriate initiation of transcription from cryptic promoters that might exist within coding regions [20], [21]. Thus SET2 serves a general function to ensure that transcription initiation by RNA pol II is properly regulated.

Phylogenetic studies of histone methyltransferases in *P. falciparum* by Cui *et al.* identified a putative ortholog of SET2 (called PfSET2) and biochemical work demonstrated that this protein possesses histone methyltransferase activity [9]. More recently, Jiang *et al.* generated a knockout of PfSET2 (also called PfSETvs) and showed that this resulted in a profound reduction of H3K36me3, providing strong evidence that PfSET2 deposits this mark on *P. falciparum* chromatin [22]. Interestingly, genome-wide analysis of the distribution of H3K36me3 found that this epigenetic mark was deposited almost exclusively at telomeres and at multi-copy gene families that undergo variant, mutually exclusive expression, for example the *var* gene family. Moreover, knockout of PfSET2 resulted in disruption of mutually exclusive expression while displaying no other detectable phenotype. Thus in *P. falciparum*, SET2 appears to be devoted to regulating mutually exclusive expression and antigenic variation. This is in sharp contrast to higher eukaryotes where SET2 deposits H3K36me3 throughout the genome, within virtually all coding regions that are actively transcribed. Given these dramatic differences in H3K36me3 distribution and function, it is unclear how PfSET2 specifically marks only very narrow regions of the parasite's genome and whether RNA pol II plays a role in its recruitment, as it does in higher eukaryotes. Understanding how PfSET2 gets targeted to genes that undergo mutually exclusive expression will shed light on a key step in the regulation of antigenic variation by *P. falciparum*.

Examination of RNA pol II from malaria parasites previously identified an unusual expansion of the CTD in parasites that infect primates when compared to rodent malaria parasites [23], however the utility of this expansion was not illuminated. In addition, a recent report suggested that epigenetic mechanisms related to antigenic variation might be significantly different when rodent parasites are compared to those of primates [24]. Given these observations, we hypothesized that the expansion of the CTD in primate parasites might be involved in mechanisms of epigenetic regulation of antigenic variation, specifically *var* gene regulation. The multi-copy *var* gene family encodes the primary antigenic determinant, PfEMP1, expressed on the surface of infected red blood cells and has been shown to be transcriptionally regulated epigenetically [25]. Using genome comparisons, chromatin immunoprecipitation, *in vitro* protein binding assays and the creation of dominant-negative transgenic lines, we confirmed that the histone methyltransferase PfSET2 is an important player in *var* gene regulation. More importantly, we identified a region of the protein that binds directly to RNA pol II *in vitro* and showed that disruption of this interaction in transgenic parasites drastically alters *var* gene expression patterns. Surprisingly, unlike the SET2 orthologs of higher eukaryotes, PfSET2 binds to RNA pol II when it is unphosphorylated, a characteristic inconsistent with its recruitment during the transcription of mRNAs and providing a possible mechanism for how recruitment of PfSET2 is limited to very narrow regions of the genome. These data suggest a model in which PfSET2 is recruited to *var* gene loci by RNA pol II during the production of previously described *var*-specific ncRNAs, and that the resulting histone mark plays a role in coordinating mutually exclusive expression and *var* expression switching.

## Materials and Methods

### Antibody production

Unmethylated, mono, di and trimethylated peptides that include and flank the H3K36 residue were synthesized and conjugated at Rockefeller University using the following epitope: RKSAPISAGI-K(Me0,1,2,3)-KPHRYRPGT. Two rabbits were immunized with the trimethylated peptides and rabbit polyclonal antibodies were obtained via a 77-day protocol (Covance). To increase specificity, the anti-H3K36me3 serum was pre-absorbed by incubating with unmethylated, monomethylated, and dimethylated peptides. 1 ml of a 1∶10 dilution of rabbit serum was incubated with 15 µl of a 1 mg/ml solution of each peptide for 30 min at 37 degrees, rocking. Specificity of the final antibody preparation was determined using peptide competition assays and verified through assaying detection of 15 ug of purified unmethylated, monomethylated, dimethylated and trimethylated peptides through slot-blot assays.

### Immunodetection of H3K36me3 in *P. falciparum* chromatin

A total of 2×10^9^ cells of the NF54 line of *P. falciparum* were lysed in RIPA buffer (Sigma; 50 mM Tris-HCl, pH 8.0, 150 mM NaCl, 1% NP-40, 0.25% sodiumdeoxycholate, 0.1% SDS), plus mammalian protease inhibitor cocktail (Sigma) supplemented with 200 mg ml-1 PMSF and 4 milligrams ml-1 pepstatin. 1 to 15 micrograms of lysates were separated by SDS-PAGE using a 15% acrylamide gel and transferred to a PVDF membrane. Membranes were blocked overnight with 5% milk. Primary antibodies were detected with horseradish-peroxidase-conjugated sheep anti-rabbit antibodies. Primary antibodies were either the anti-H3K36me3 described above or commercially available antibodies against H3-core.

### Extraction and purification of histones from *P. falciparum*

*P. falciparum* histones were extracted and purified by a modified protocol as previously described by Schecter, *et al.* [26]. Briefly, infected NF54 cells were lysed by saponin and released parasites were washed twice in 1× PBS. The parasite cell pellet was resuspended in 1 mL of ice cold water to induce lysis and incubated for 30 min on a rotator at 4°C. The nuclei was pelleted by spinning at 10,000 g for 10 min at 4°C. The supernatant was discarded and the pellet was resuspended in 0.4 N H2SO4 and incubated overnight on a rotator at 4°C. The sample was then spun down for 20 min at 14,000 g. The supernatant was dialyzed against H_2_O for 2 h to overnight. The sample was retrieved and lyophilized in a SpeedVac overnight. The powder was diluted in ∼100 µL H_2_O. SDS-PAGE/Western was performed to detect the H3K36me3 mark.

### Chromatin immunoprecipitation

Chromatin Immunoprecipitation was performed as previously described [5], [27] using samples prepared from synchronized ring stage parasites from recently cloned lines with established *var* expression profiles [28]. Pre-absorbed anti-H3K36me3 antibodies (see above), anti-H3 core histone (positive control) or no antibody (negative control) were used for the immunoprecipitation. Isolated DNA was then analyzed by quantitative RT-PCR. Graphs display the ratio of the relative quantity of bound DNA divided by the total input (bound+unbound), normalized to a control gene (PfCTRP).

### Expression and purification of *P. falciparum* and *P. berghei* recombinant CTD of Rpb1

Genomic DNA from *P. falciparum* and *P. berghei* were used as template for PCR amplification of the R2 and R3 regions (R2R3) of the C-terminal domain (CTD) of Rpb1. The sequence encoding a 3× Flag-tag was incorporated into the reverse primer in each reaction. The amplified products were cloned into the pSUMO expression vector, which includes an N-terminal His_6_ tag. Recombinant proteins were expressed in BL21(DE3) *E. coli* cells, which were subsequently purified using Nickel-nitrilotriacetic acid (Ni-NTA) beads (Qiagen). Proteins were concentrated and stored in 50 mM Tris, pH 8.0, 0.1 M NaCl and 10% glycerol. Primers used for PCR amplification are described in Table S1.

### Expression of recombinant PfSET2 fragments and the SET2-Rpb1 interacting (ySRI) domain from yeast

The DNA sequences encoding five different PfSET2 fragments (PfSET2_(1–359)_, PfSET2_(351–828)_, PfSET2_(1060–1535)_, PfSET2_(1540–2080)_ and PfSET2_(2264–2548)_) were amplified by PCR using *P. falciparum* NF54 genomic DNA as template. The DNA sequence encoding the ySRI was amplified by PCR using *Sacharomyces cerevisiae* genomic DNA. The sequence encoding an HA epitope tag was incorporated into the reverse primers to create HA-tagged versions of each fragment. The sequence of the PCR primers is provided in Table S1. The PCR products were cloned into pSUMO and protein expression induced in BL21(DE3)RIL *E. coli* cells as described above. Cultures expressing the recombinant proteins were harvested and lysed as described above, with the exception that EDTA and MgCl_2_ were added to the lysis buffer to final concentrations of 0.5 mM and 1.0 mM respectively. Cleared lysates were used for co-immunoprecipitation assays and expression of truncates were monitored by SDS-PAGE and Western blot.

### In vitro phosphorylation of the CTD of Rpb1

Purified Flag-tagged R2R3 portions of the CTD of Rpb1 from *P. falciparum* and *P. berghei* were phosphorylated using Cdc2 kinase (NEB). Phosphorylation of the CTD was monitored by SDS-PAGE and Western blot.

### Co-Immunoprecipitation assays

Lysates from bacterial cultures expressing HA-tagged PfSET2 fragments and the ySRI were incubated with purified, recombinant Rpb1 CTD in both phosphorylated and unphosphorylated forms in 3% BSA at 4°C for 16 h. Reactions were precleared on control agarose resin (Thermoscientific) and loaded onto Protein A agarose beads (Millipore) for two hours. Bead were washed extensively three times with 50 mM Tris, pH 8.0, 0.5 M NaCl, 0.5% Tween-20 and 10% glycerol. Polyclonal anti-HA antibody (Rabbit; Sigma) was used to immunoprecipitate PfSET2 and any interacting proteins. Immunoprecipitated proteins were separated by SDS-PAGE and the CTD of Rpb1 was detected by Western blotting using anti-Flag antibody.

### SDS-PAGE and western blotting

Proteins were separated on 4–20% Tris-HCl acrylamide gels and analyzed by Western blot. Antibodies were detected using the SuperSignal West Pico Chemiluminescent substrate reagents from Thermoscientific. Monoclonal mouse anti-HA and anti-His antibodies (Sigma) were used at dilutions of 1∶2000 and 1∶6250 respectively. Monoclonal anti-Flag (Sigma) was used at a dilution of 1∶4000. All antibodies were detected using anti-mouse IgG (whole molecule) peroxidase, produced in rabbit (Sigma) at a dilution of 1∶100,000.

### Creation of dominant-negative transgenic parasite lines

The dominant negative experiments utilized clonally stable NF54 bulk cultures previously generated by Frank *et al.* [28]. Two of NF54 subclones called A3 and C3 were cultivated and transfected as described by Dzikowski, *et al.* [29]. Expression constructs were created using the PLN-ENRGFP plasmid [30]. For the dominant-negative version of PfSET2, the sequence encoding the N-terminal 359 amino acids was fused to a C-terminal HA tag. A control construct included the sequence encoding Firefly luciferase. Both dominant-negative and control parasite lines were initially selected by culturing in the presence of 2 µg/mL blasticidin S HCL for 42 days. Portions of each culture were then transferred to new flasks and grown in the presence of 10 µg/mL blasticidin for 49 days. The 2 µg/ML blasticidin cultures were maintained throughout the duration of the experiment.

### RNA extraction and cDNA synthesis

RNA was extracted as previously described [29]. Briefly, RNA was TRiZol (invitrogen) extracted from synchronized late ring stage parasites grown at both 2 and 10 µg/mL blasticidin and purified on PureLink (invitrogen) columns following manufacturer's protocol. Purified RNA was treated with Deoxyribonuclease I (DNAse I) (Invitrogen). cDNA was synthesized from approximately 800 ng of RNA in a reaction that included Superscript II RNase H reverse transcriptase (invitrogen) with random primers (Invitrogen) as described by the manufacturer.

### Quantitative RT-PCR

For chromatin immunoprecipitation studies, forward and reverse primer pairs with complementary sequences within the *var* gene PF3D7-0421100 were used for qRT-PCR. The standard curve method was used to quantitate mRNA levels (ABI user manual). All reactions were compared to control genes encoding acyl-tRNA synthetase, actin and a *P. falciparum* circumsporozoite protein. All reactions were performed using ITAQ SYBR supermix (Bio-Rad) in 10 uL reactions using an ABI Prism 7900 HT real time PCR machine. For gene expression studies, cDNA was evaluated for genomic DNA contamination prior to assessing *var* gene expression. To detect transcription of all *var* genes, we employed a real-time PCR set developed by Salanti *et al.* [31] and modified by Dzikowski [32] and Frank [28]. To detect Pfset2 or specifically the SRIR domain included in dominant negative constructs, specific primers were designed and are shown in Table S2. ΔCT values for each individual primer pair were obtained by subtracting the CT value from the CT value of the control seryl-tRNA synthetase (User bulletin 2, Applied Biosystems, http://www.appliedbiosystems.com). The formula 2^ΔCT^ was used to convert the ΔCT values to relative copy numbers. All reactions were performed in duplicate and averaged in Microsoft Excel. Transcriptional profiles of all the individual *var* genes are presented as pie graphs prepared as described by Frank *et al.* [28]. The most highly expressed *var* gene was defined as the dominant gene.

## Results

### Identification of a histone methyltransferase/demethylase pair specific to primate malaria parasites

Previous examination of the CTD of RNA pol II from numerous primate and non-primate *Plasmodium* species found that the CTD is uniquely expanded in parasites that infect primates [23]. In higher eukaryotes, this domain contains repeats which actively recruit various proteins to the RNA pol II enzyme complex. Of particular interest for our studies, when appropriately phosphorylated these repeats directly bind histone modifiers, recruiting them to the actively transcribing polymerase complex and thus marking recently transcribed regions of the genome [33]. Together these observations led us to hypothesize that primate parasites might possess one or more unique histone modifiers when compared to rodent parasites, and that such modifiers would directly interact with the expanded CTD of RNA pol II.

**Figure 1:**
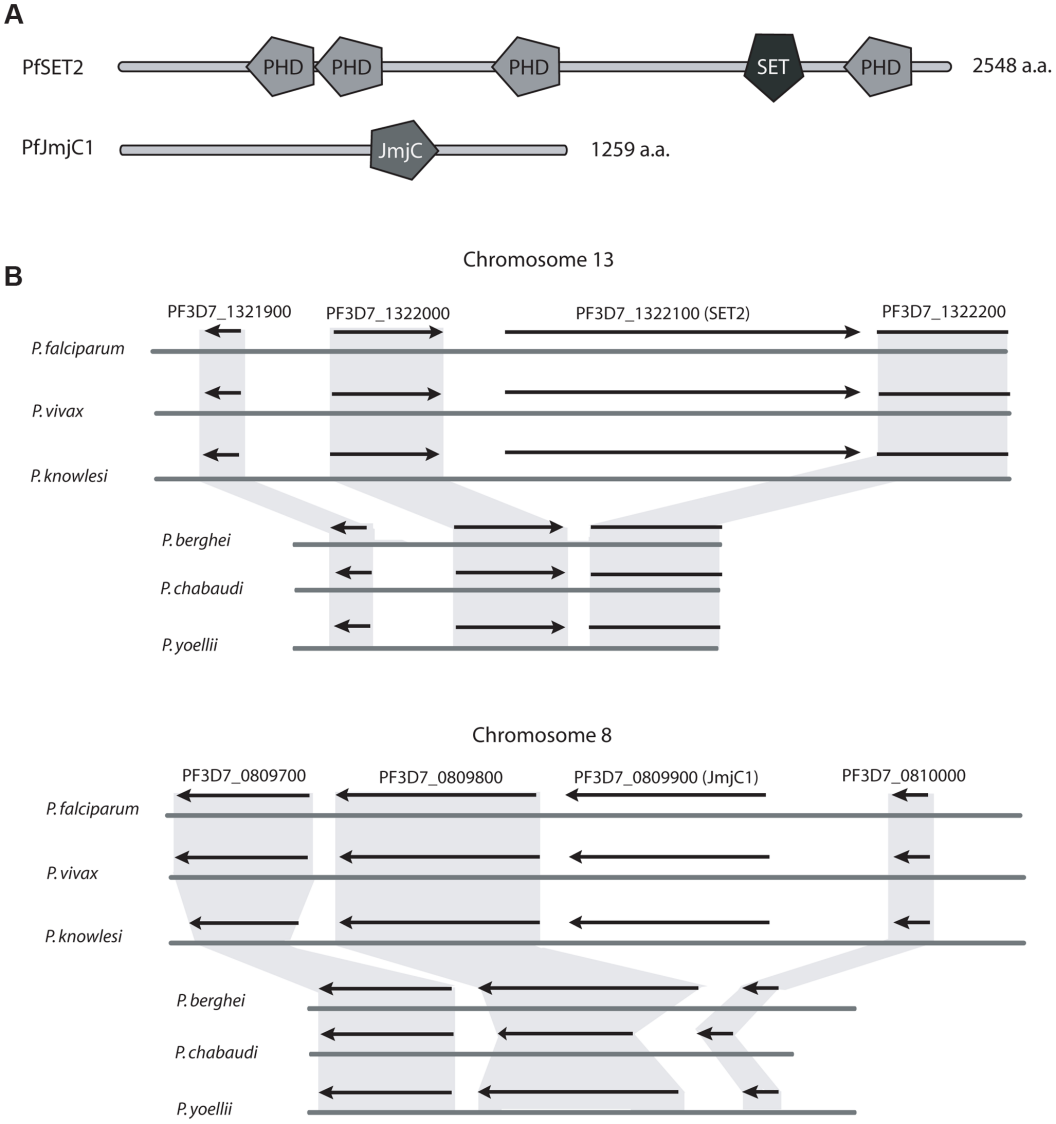
Two histone modifiers unique to primate parasites. A comparative screen of the genomes of primate and rodent malaria parasites identified two histone modifiers unique to *P. falciparum*, *P. vivax* and *P. knowlesi*, SET2 and JmjC1. (A) Domain searches of the amino acid sequences using SMART (Simple Modular Architecture Research Tool, http://smart.embl-heidelberg.de) identified several domains typical of chromatin modifying enzymes, including several PHD domains and a SET2-like methyltransferase domain in PfSET2 and a predicted JmjC type demethylase in PfJmjC1. (B) Syntenic regions of chromosomes 13 and 8 of three primate parasites and three rodent parasites. Extensive synteny is observed throughout these chromosomal regions, with the exception of SET2 and JmjC1, which are missing from the genomes of the rodent parasites. The flanking genes are highly conserved. Figure adapted from Plasmodb.org.

As a first step to test this hypothesis, we compared the genome sequences of three primate parasites (*P. falciparum*, *P. vivax* and *P. knowlesi*) with the genomes of three rodent parasites (*P. berghei*, *P. yoellii*, and *P. chabaudi*). Specifically, we used a collection of 202 transcription factors and histone modifiers identified in *P. falciparum* by Bischoff *et al.* [34] to perform a comparative screen of proteins encoded within the genomes of the other five *Plasmodium* species. Three protein encoding genes were identified that were present within the genomes of *P. falciparum*, *P. knowlesi* and *P. vivax* but missing in all three rodent lineages. These included the histone methyltransferase *PfSet2* (PF3D7_1322100) and its complementary demethylase *PfJmjC1* (PF3D7_0809900) (Figure 1A). These two modifiers are predicted to add and remove methyl groups from the lysine in the 36^th^ position of histone H3 (H3K36) respectively, thus suggesting they represent a complementary pair of histone modifiers specific to primate parasites. Of particular interest for this study, SET2 orthologs from higher eukaryotes have been shown to directly bind to the CTD of RNA pol II, adding confidence to our hypothesis that the presence of these histone modifiers might be directly related to the expanded CTD found in primate parasites. The third gene found to be unique to primate parasites, a predicted zinc-finger transcription factor expressed in gametocytes (PF3D7_1134600), will not be described further here.

To verify that orthologs of *Set2* and *JmjC1* are in fact absent from the rodent parasite genomes and not simply mis-annotated or not identified due to low levels of sequence similarity, we examined the genome assemblies for syntenic open reading frames that could represent the missing genes. All six genomes displayed near perfect synteny in the regions surrounding both *PfSet2* and *PfJmjC1*, however these two genes are entirely missing from the genomes of all three rodent parasites (Figure 1B). No predicted pseudogenes or other similar genome features were found where these genes were predicted to reside, suggesting that insertion/deletion events define the primate vs rodent lineages at these positions in the genome. As final confirmation that the genome assemblies are correct, PCR primers were designed to the regions of the *P. berghei* genome flanking the syntenic positions where *Set2* and *JmjC1* were predicted to exist. PCR amplifications yielded the predicted product in both cases and confirmed that neither *Set2* nor *JmjC1* exist at these positions in the genome (not shown). Degenerate PCR primers to *Set2* and *JmjC1* also failed to amplify products using *P. berghei* genomic DNA as template, further suggesting that these two genes are likely not present within the rodent parasite genomes.

### Detection of H3K36me3 in *P. falciparum* chromatin

Methyltransferases of the SET2 class are predicted to transfer three methyl groups to histone H3 at the lysine in the 36^th^ position (H3K36me3) [14]. If PfSET2 possesses this activity, H3K36me3 should be found within the chromatin of *P. falciparum*. Conflicting reports exist in the literature regarding the presence of this particular histone modification. Issar and colleagues were unable to detect H3K36me3 using a commercially available antibody preparation generated against yeast H3K36me3 [35], and Trelle *et al.* also did not detect this modification in a proteomic analysis of chromatin extracted from cultured malaria parasites [36]. In contrast, using a different antibody preparation, Cui *et al.* detected H3K36me3 within *P. falciparum* chromatin and also detected the appropriate methyltransferase activity using recombinant PfSET2 on H3 core [37]. More recently, Jiang *et al.* detected H3K36me3 using an alternative source of commercially available antibodies, and found that the level of this modification was greatly reduced with PfSET2 was knocked out, thus provided more convincing data for the presence of this histone modification in *P. falciparum* and its deposition by PfSET2 [22]. Nevertheless, *P. falciparum* histone H3 has several amino acid polymorphisms close to K36 when compared to higher eukaryotes, making the use of commercially available antibodies generated against H3 peptides from heterologous organisms potentially problematic, and providing a possible explanation for the disparate results. Thus independent confirmation of the presence or absence of H3K36me3 within chromatin from *P. falciparum* was desirable.

**Figure 2:**
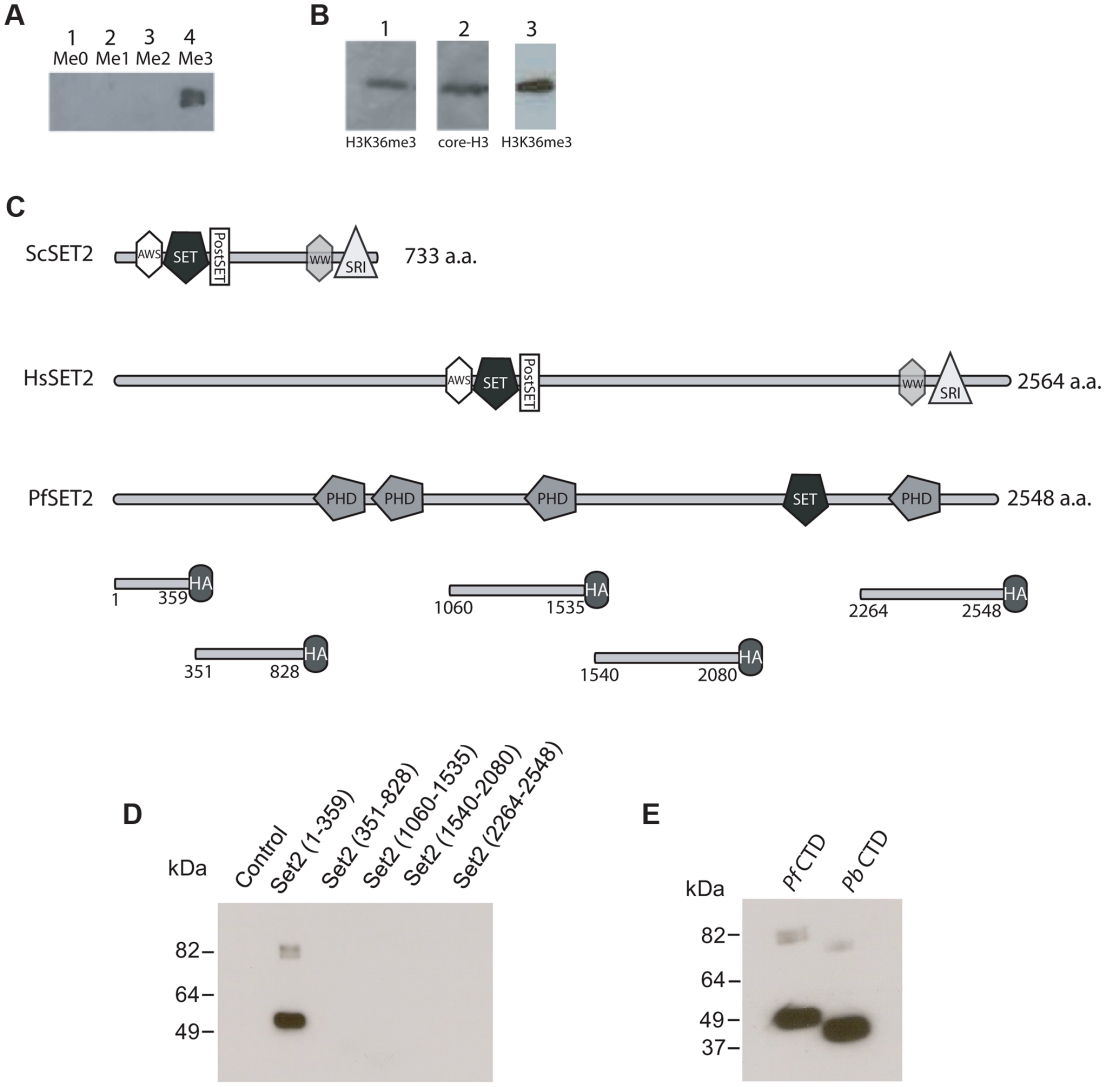
Detection of H3K36me3 in chromatin from *P. falciparum* and identification of the Rpb1binding region of PfSET2. (A) Slot blots of the synthetic peptide sequence RKSAPISAGIKKPHRYRPGT. This sequence includes K36 of histone H3 of *P. falciparum* (underlined). Lane 1, unmethylated peptide. Lane 2, peptide synthesized with a mono-methyl group at the K36 position. Lane 3, peptide synthesized with a di-methyl group at the K36 position. Lane 4, peptide synthesized with a tri-methyl group at K36 position. All lanes included 15 µg of purified peptide. The dot blot was probed with antibodies against the tri-methylated form of the peptide and demonstrated that the antibodies are specific to the tri-methylated form. (B) Western blot of *P. falciparum* protein extracts taken from early trophozoites. Lane 1, blot of total parasite proteins probed with anti-H3K36me3 antibodies. Lane 2, blot of total parasite proteins probed with anti-H3-core antibodies. Both antibodies recognize bands migrating at ∼18 kDa. Lane 3, blot of acid extracted histones obtained from cultured parasites and probed with anti-H3K36me3 antibodies. (C) Comparison of the architecture of SET2 orthologs from *S. cerevisiae*, *H. sapien* and *P. falciparum*. Note that the SET2-Rpb1-Interacting domain (SRI) is found at the C-terminal end of both the yeast and human SET2 proteins. The protein domains shown in the figure are displayed using the format derived from SMART (Simple Modular Architecture Research Tool, http://smart.embl-heidelberg.de). Below PfSET2 are displayed the portions produced as HA-tagged, recombinant proteins for use in co-immunoprecipitation assays. The numbers below each fragment represent the amino acid positions within the full-length protein. The tandem PHD domains (amino acids 829–1060) were not included because the DNA encoding the this region of the protein was unstable in the expression vector. The SET domain was also not included because the structure and function of this domain is conserved and was previously shown not to bind the CTD in yeast and human systems. (D) Co-immunoprecipitation assays used to identify the region of PfSET2 that interacts with the CTD of Rpb1. Flag-tagged fragments of the CTD of Rpb1 shown in E were incubated with bacterial lysates containing the truncates of PfSET2 shown in C. Immunoprecipitation was performed using anti-HA beads followed by SDS-PAGE and Western blotting using anti-Flag antibodies. Only the most N-terminal portion of PfSET2 (amino acids 1–359) was found to interact with the CTD of Rpb1. The control reaction consisted of an HA-tagged PHIST protein similarly incubated with the CTD of Rpb1. (E) The N-terminal region of PfSET2 also binds to Rpb1 regions with fewer numbers of repeats. Co-immunoprecipitation assay performed as in D, using either the CTD of Rpb1 from *P. falciparum* (left lane) or *P. berghei* (right lane). The *P. berghei* CTD has only 8 heptad repeats.

Given the identification of PfSET2 as a potentially unique histone modifier in primate parasites, a first step was to definitively determine if the predicted histone modification was actually present within the chromatin of primate parasites. Due to the conflicting results obtained using antibodies generated against H3K36me3 peptides from other organisms, we generated custom antibodies using synthetic peptides of *P. falciparum* histone H3. To determine the ability of these antibodies to distinguish between H3K36, H3K36me, H3K36me2 and H3K36me3, we utilized slot-blots of synthetic peptides in all four methylated states. Our custom antibodies only recognized H3K36me3 (Figure 2A), thus providing us with a reagent to determine if this modification is present in chromatin extracted from cultured *P. falciparum*. Western blots of total protein extracts and of acid extracted histones obtained from cultured parasite detected a band of appropriate size that co-migrated with a band detected by antibodies against core H3 (Figure 2B). Immunofluorescence assays of fixed parasites also detected H3K36m3 within parasite nuclei (Figure S1). These data, combined with the H3 methyltransferase activity previously detected *in vitro* by Cui *et al.* [9] as well as the recent knockout studies [22] suggest that PfSET2 is in fact a bona fide H3K36 methyltransferase.

### Unique architecture of PfSET2 and binding to RNA pol II

Investigations of SET2 orthologs from both yeast and humans have identified a conserved architecture in which the methyltransferase domain is found near the N-terminus of the protein [16], [17] (Figure 2C). The RNA pol II CTD binding region, referred to as the SET2-Rpb1-interacting (SRI) domain, is located within the C-terminal region of the protein. The SET2 protein from *P. falciparum* has a very different architecture, however [38]. The putative methyltransferase domain is found near the C-terminus, and the remainder of the protein does not possess any regions with significant similarity to the SRI domains identified in the human or yeast SET2 proteins (Figure 2C). In addition, PfSET2 possesses several PHD domains (domains known to be involved in protein-protein interactions and epigenetic regulation) that are not found in the SET2 proteins of either yeast or mammals (Figure 2C). These differences prevented the computational identification of a potential SRI-like domain within PfSET2, thus if or how PfSET2 is recruited by RNA pol II remained unknown, and we could not rule out the possibility that PfSET2 does not bind to the RNA pol II complex at all but rather is recruited to specific regions of the genome via an alternative mechanism. Similarly, the recent finding that the H3K36me3 mark is not found throughout the genome where RNA pol II actively transcribes mRNA further suggested that PfSET2 is not recruited in a similar manner to what has been described in higher eukaryotes.

Our original identification of PfSET2 was based on its hypothetical interaction with the extended CTD of Rpb1 from primate malaria parasites. Therefore, we attempted to determine if such an interaction actually exists. To determine if PfSET2 can interact with the CTD of Rpb1 from *P. falciparum*, we adopted the *in vitro* protein binding assay developed to identify the SRI in both human and yeast SET2 [16], [17]. In these assays, recombinantly produced, epitope tagged portions of SET2 were combined with the CTD that was tagged with an alternative epitope. After incubation in the appropriate binding buffer, co-immunoprecipitation (co-IP) assays were performed to identify the regions of PfSET2 that stably interact with the CTD. Five different fragments covering the entire length of PfSET2 (with the exception of the two tandemly arranged PHD domains and the methyltransferase region) (Figure 2C) were produced recombinantly in *E. coli* and incubated with the R2/R3 regions of RNA pol II from *P. falciparum* (Figure S2) [23]. These assays found that the first 359 amino acids of the N-terminus of PfSET2 bound robustly to the CTD of PfRpb1, whereas no other region of the protein displayed any detectable binding activity (Figure 2D). We hereafter refer to this region of PfSET2 as the SET2-Rpb1-interacting region (PfSRIR). The SRI domain from yeast SET2 does not require the entire CTD for efficient binding and can also interact with a substantially truncated portion of the CTD repeat region [17], as observed in both *in vitro* assays and crystal structure analysis [39]. We similarly found that the PfSRIR does not require the entire CTD for binding and can also interact with a substantially shorter CTD from *P. berghei* (Figure 2E). These experiments show that despite the lack of any detectable similarity in amino acid sequence or domain architecture, PfSET2 does indeed bind to the CTD of Rpb1, suggesting that it is similarly recruited to specific regions of the genome through the action of RNA pol II.

### Binding of PfSET2 to the CTD of RNA Pol II is disrupted by phosphorylation

Binding of SET2 to Rpb1 in humans and yeast has been shown to be dependent on phosphorylation of the serine residues found within the heptad repeats of the CTD [16], [17]. All higher eukaryotic CTDs consist of a tandem array of the conserved heptad YSPTSPS/K. The human CTD contains 52 heptads, whereas the CTD of the yeast *S. cerevisiae* has 26 [40]. During the act of transcription, the CTD undergoes changes in the phosphorylation status of the serines at positions 2 and 5 of the heptads, thereby altering the conformation of this domain and changing which proteins are recruited by the CTD to the protein complex [41]. Phosphorylation at positions 2 and 5 of the heptads occurs when the polymerase is engaged in transcriptional elongation of mRNAs, and this specific modification has been shown to recruit SET2 in humans and yeast [18], [19]. This property directly ties SET2 recruitment to mRNA elongation, thus in the genomes of these organisms, H3K36me3 is found within the body of most transcribed genes.

**Figure 3:**
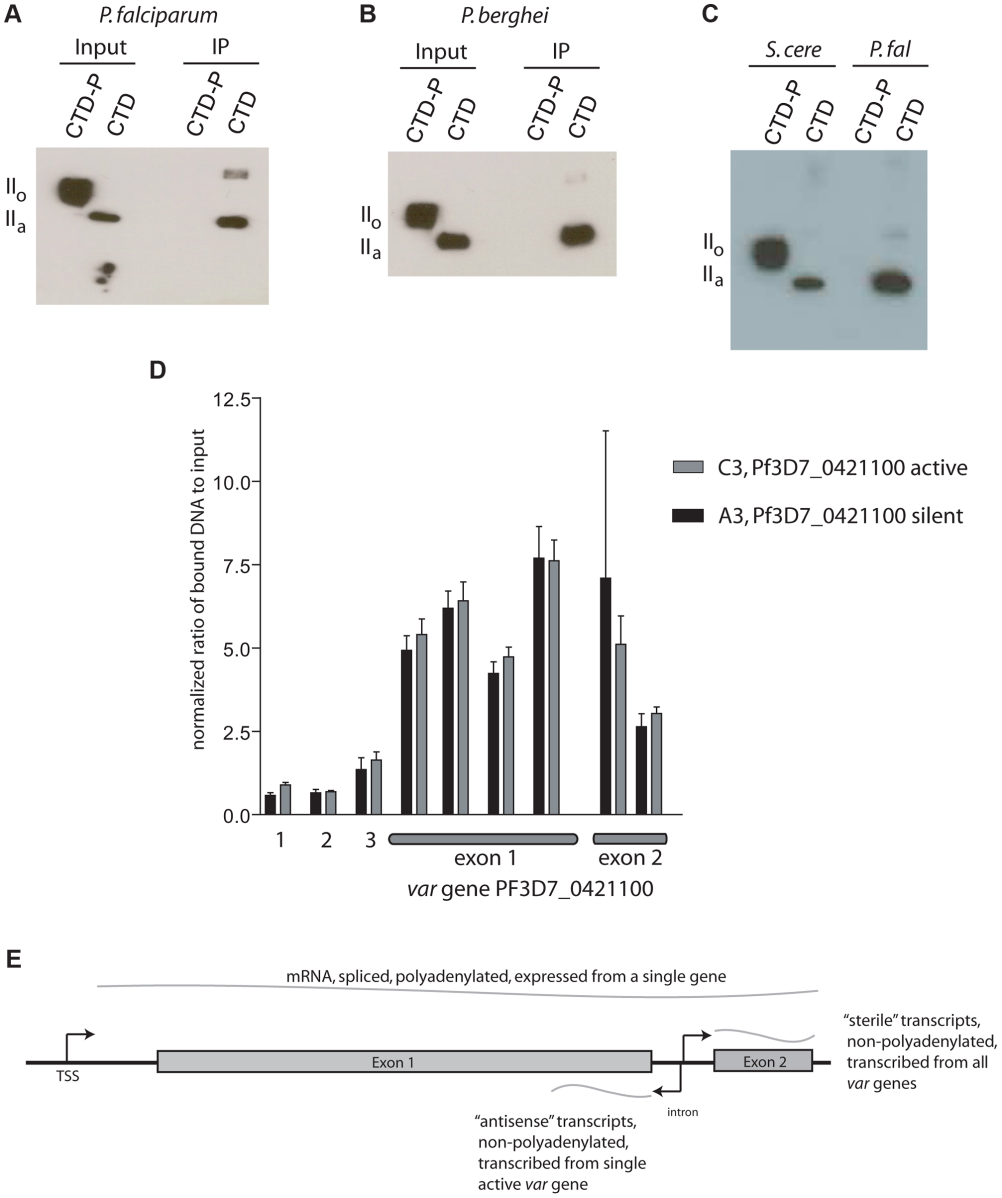
Phosphorylation dependence of PfSET2 binding to the CTD and enrichment of H3K36me3 at *var* loci. (A) Co-immunoprecipitation assays showing that phosphorylation of the CTD of Rpb1 eliminates binding by PfSET2. The two left lanes show the flag-tagged *P. falciparum* Rpb1 in its phosphorylated (II_o_) or unphosphorylated (II_a_) states. The right two lanes show blots of co-immunoprecipitation assays after incubation of bacterial lysates containing the HA tagged, N-terminal region of PfSET2 (PfSET2_1–359_) with phosphorylated and unphosphorylated CTD. Phosphorylation of the CTD disrupts binding by PfSET2. (B) Co-immunoprecipitation assays as in A performed with the CTD of Rpb1 from *P. berghei*. (C) Direct comparison of the CTD binding affinities of SET2 from *S. cerevisiae* and *P. falciparum.* The SRI from *S. cerevisiae* (left two lanes) interacts preferentially with the phosphorylated form of the *P. falciparum* CTD (left) but also displays lesser binding to the unphosphorylated form (right). The right two lanes show the same assay with the *P. falciparum* SRIR region (amino acids 1–359). The SRIR displays specific binding to the unphosphorylated CTD (right) and little or no binding to the phosphorylated form (left).(D) Chromatin immunoprecipitation assays from ring stage parasites using anti-H3K36me3 antibodies. The assays show enrichment of the H3K36me3 mark in both exons of *var* gene PF3D7_0421100. Lanes 1 and 2 represent control genes encoding seryl t-RNA synthetase and actin, respectively. Lane 3 represents the gene for circumsporozoite protein. The remaining lanes represent regions within the coding portion of both exons of the *var* gene PF3D7_0421100. Black bars show results from chromatin extracted from the C3 line of NF54, in which PF3D7_0421100 is the actively expressed *var* gene. The gray bars show chromatin extracted from the A3 line of NF54 in which this *var* gene is transcriptionally silent. The bars display the mean +/− standard deviation of relative amounts of bound DNA (see methods) from four independent experiments. Similar experiments using no-specific antibodies or control antibodies against core histone H3 showed no specific enrichment at PF3D7_0421100 (not shown). The graph shown represents the signal for each gene normalized to the control gene *ctrp*. The data is shown as % input without normalization in Figure S7. (E) Schematic representation of a typical *var* gene. Note that each gene has three promoters, one upstream of the coding region and responsible for transcribing an mRNA from the single, active *var* gene and two within the intron transcribing ncRNAs in opposite directions. The intron promoter that transcribes the antisense ncRNA is active during rings stages and is only active in genes that are also transcribing mRNA. The intron promoter transcribing ncRNA in the sense direction leads to expression of “sterile”, ncRNAs during late trophozoites and schizonts and is active in most or all members of the *var* gene family.

Given that Rpb1 of *P. falciparum* possesses very similar heptad repeats as found in model eukaryotes, including the serines at positions 2 and 5 that serve as phosphorylation sites (Figure S2), we anticipated that PfSET2 would similarly require phosphorylation for binding to the CTD. However, our initial experiments detected robust binding of PfSET2 to the unphosphorylated CTD (Figure 2D), suggesting that this interaction might be fundamentally different in malaria parasites. To further determine if phosphorylation of the CTD influences binding by PfSET2, we exposed recombinant CTD to Cdc2 kinase, a kinase known to phosphorylate the serine residues within CTD heptads, then repeated our co-IP assay. Surprisingly, phosphorylation of the CTD completely disrupted PfSET2 binding (Figure 3A and B).

The affinity of PfSET2 for an unphosphorylated CTD and disruption of binding by phosphorylation is fundamentally opposed to the current model of SET2 recruitment as described for yeast and mammals. If true, this might suggest a very different role for PfSET2 in parasite biology. Given the potential significance of this finding, we chose to more rigorously explore this interaction by directly comparing binding affinities of the yeast SRI (ySRI) to that of the PfSRIR. Recombinant ySRI and PfSRIR were incubated with both the unphosphorylated and phosphorylated versions of the *P. falciparum* CTD and co-IP assays were performed side-by-side. In agreement with previous reports [17], [18], the ySRI displayed a robust affinity for the phosphorylated CTD, with much weaker binding to the unphosphorylated form also evident (Figure 3C). This minor binding to the unphosphorylated *Plasmodium* CTD is somewhat different from what was observed for the yeast CTD and might reflect subtle differences in the heptad repeat structure between the two organisms [23]. In stark contrast, the PfSRIR only bound the unphosphorylated CTD, thus displaying the opposite affinity and much greater specificity than the ySRI. These results were unexpected and suggest that if Rpb1 of *P. falciparum* undergoes phosophorylation of its CTD heptads similarly to all other studied eukaryotes, PfSET2 might not interact with the RNA pol II complex when it is actively transcribing mRNA, but rather while it is engaged in an alternative function.

### H3K36me3 marks both active and silent *var* genes

If binding of PfSET2 to the RNA pol II complex requires phosphorylation of the serines of the CTD of Rpb1 as has been shown in higher eukaryotes, we anticipated that the H3K36me3 mark would be found at actively transcribed genes within the parasite's genome, including most or all housekeeping genes. However, our *in vitro* data suggested that PfSET2 binds to RNA pol II when the CTD is unphosphorylated, suggesting that it might be recruited when the polymerase complex is engaged in a function other than transcribing an mRNA. To investigate these potentially contradictory models, we performed chromatin immunoprecipitation (ChIP) experiments to examine the deposition of H3K36me3 in live parasites utilizing the custom antibodies generated against H3K36me3. Following standard ChIP procedures, we isolated chromatin from cultured asexual parasites and performed quantitative real-time PCR on the immunoprecipitated fractions. We examined two “housekeeping” genes that are known to be actively transcribed into mRNA as well as a stage specific gene (*csp*) that is transcriptionally silent during asexual development [42], [43]. In addition, we investigated the deposition of H3K36me3 at *var* genes. These genes serve as a particularly useful model since they are subject to both silencing and activation through epigenetic mechanisms [25]. Thus, it is possible to examine individual *var* genes when they are “on” and actively transcribing mRNA or when they are “off” and no mRNA synthesis can be detected. We performed parallel ChIP experiments using different isogenic parasite lines that had been grown for a limited number of generations after cloning and that expressed different subsets of the *var* gene family. This enabled us to examine individual *var* genes in both their active and silent states.

The ChIP data found very little or no H3K36me3 at the two actively transcribed housekeeping genes or at the transcriptionally silent *csp* locus (Figures 3D and S7), suggesting that this mark is not simply associated with recently transcribed regions of the genome. However the mark was easily detectable within the coding regions of *var* genes. More importantly, we found this mark to be equally present within the coding regions of *var* genes regardless of whether they were active or silent. Similar results were recently reported by Jiang *et al.* using a genome-wide analysis of H3K36me3 deposition [22]. The absence of H3K36me3 at actively transcribed housekeeping genes and its presence at both silent and active *var* genes implies that, unlike in yeast and mammals, H3K36me3 is not a universal mark for actively transcribed chromatin and further suggests that PfSET2 is not recruited by RNA pol II when it is actively synthesizing mRNA. The disruption of the *in vitro* PfSET2/Rpb1 interaction by phosphorylation similarly suggests an interaction unrelated to mRNA synthesis. However, RNA pol II has been shown to transcribe ncRNAs from *var* introns when the genes are both actively expressed and when they are silent (Figure 3E) [27], [44], thus providing an alternative opportunity for the recruitment of PfSET2 to *var* genes that is independent of mRNA synthesis. ncRNA production at *var* loci therefore provides a possible alternative opportunity for RNA pol II to recruit PfSET2, and would be consistent with the presence of this mark at both active and silent *var* loci.

### Over-expression of a dominant-negative version of PfSET2 induces profound changes in *var* gene expression

The multi-copy *var* gene family encodes the variant surface antigen PfEMP1 [45]–[47]. The family includes approximately 60 *var* genes expressed mutually exclusively, with the transcriptionally active copy determining the antigenic and virulence phenotype of the infected RBC. Once activated, a *var* gene tends to remain active for many replicative cycles and switching is detectable only after lengthy time periods in cultured parasites [28], [48], [49]. This reflects the low transcriptional switching rate that is typical of this gene family and that is thought to be required for the efficient antigenic variation needed to maintain a long-term infection within the human host. The detection of H3K36me3 at *var* loci suggested that PfSET2 might play a role in *var* gene transcriptional regulation, and parasites in which PfSET2 was knocked out displayed a complete loss of mutually exclusive *var* gene expression, thus confirming the importance of PfSET2 in *var* gene regulation [22]. However, how PfSET2 is specifically recruited to *var* gene loci and a possible role for RNA pol II in its recruitment remained unresolved. If PfSET2 is recruited to *var* genes through its interactions with the RNA pol II complex as theorized here, disruption of this interaction could lead to changes in *var* gene expression. We aimed to test this model by disrupting PfSET2/RNA pol II interactions in transgenic parasites.

**Figure 4:**
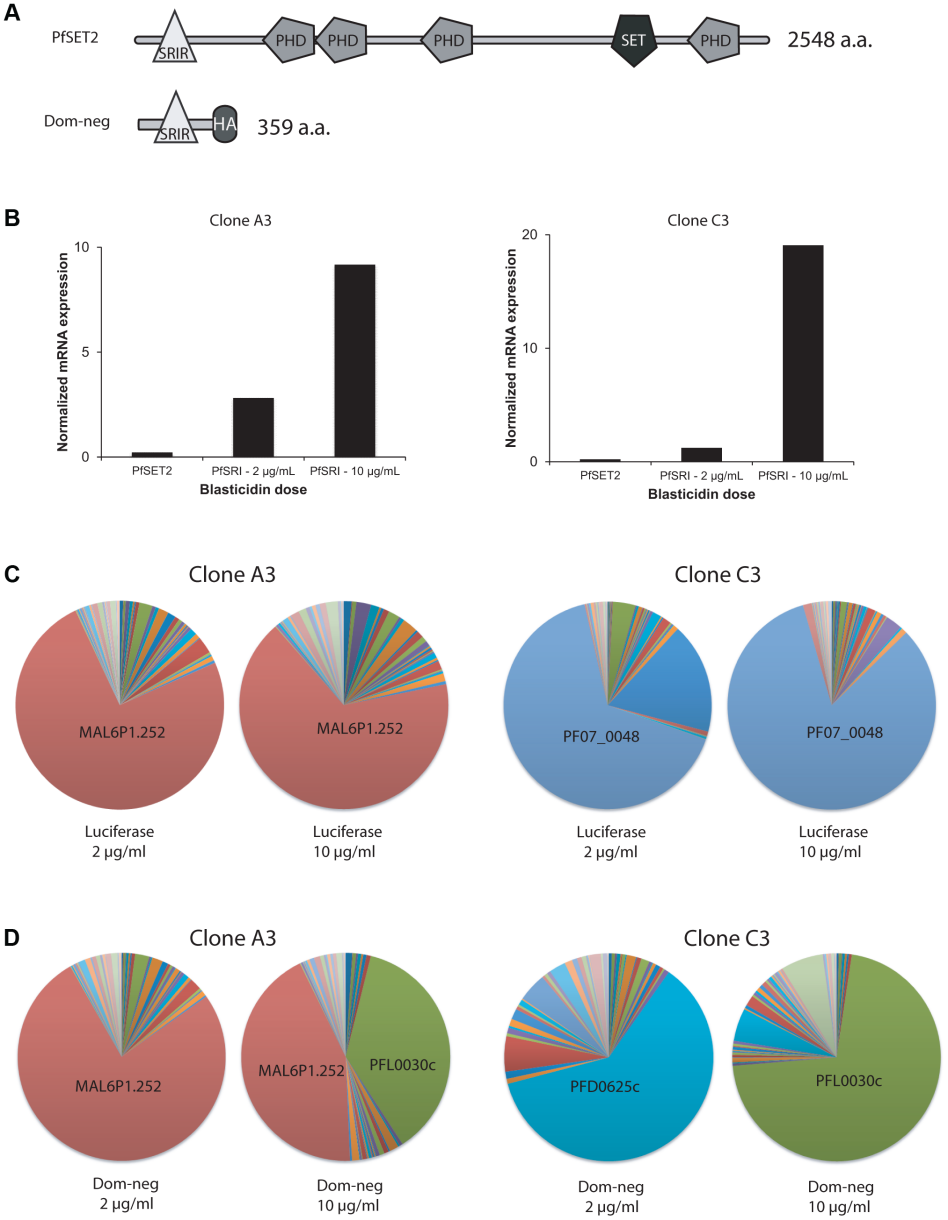
Over-expression of a dominant-negative version of PfSET2 in cultured parasites. (A) Schematic diagrams showing the domain structures of the endogenous PfSET2 protein (top) and the truncated, dominant-negative version that contains only the N-terminal 359 amino acids (bottom), including the CTD-binding domain (SRIR). (B) Histograms showing RNA expression levels of the endogenous PfSET2 gene (PfSET2) and the dominant-negative construct (PfSRIR) under 2 and 10 µg/ml blasticidin. Two separate subclones of NF54 were transfected, A3 (left) and C3 (right). (C and D) Pie charts representing *var* gene expression patterns for A3 (left) and C3 (right) when over-expressing either firefly luciferase (C) or the PfSET2 dominant-negative construct (D). The different wedges of the pie indicate the proportion of the total *var* mRNA pool represented by transcripts for each individual gene (as described in [28]).

In Figure 2, we identified the PfSRIR by its ability to bind to the CTD of Rpb1 *in vitro* and theorized that this binding activity mediates its recruitment to the RNA pol II complex. This region is found within the most N-terminal 359 amino acids and is relatively distant from other portions of the protein that contain recognizable motifs (Figure 4A). If this portion of the protein is sufficient to mediate CTD binding as observed in the *in vitro* assay, a truncated version of the protein expressed in transgenic parasites containing only this 359 amino acids could potentially compete with endogenous PfSET2 for binding to Rbp1. Since the truncated version of the protein is enzymatically dead, this could result in a “dominant-negative” phenotype, potentially altering or disrupting *var* gene regulation. While the phenotype of a dominant negative is typically less pronounced than a knockout, this method has the advantage of enabling the investigator to probe the importance of specific regions or domains of the protein being studied, thus revealing mechanistic insights not apparent with a knockout. In this case, we were interested in testing the hypothesis that over-expression of the PfSRIR alone will result in an alteration in *var* gene expression, presumably by affecting the ability of RNA pol II to recruit PfSET2. We expected the phenotype to be less severe than what was observed for the PfSET2 knockout, particularly since the endogenous PfSET2 gene remained unaltered and fully active, with only its recruitment by the RNA pol II complex affected.

To over-express the PfSRIR of PfSET2, we cloned a fragment of the gene encoding the first 359 amino acids into the vector pLN-ENRGFP [30]. This vector expresses the gene of interest under control of the calmodulin promoter while also expressing the *blasticidin S deaminase* (*bsd*) gene to enable selection of transfected parasites. The use of *bsd* as a selectable marker enables control of transgene expression levels by manipulating drug concentrations in the culture media [50]. At low concentrations of blasticidin (2 µg/ml), a low but detectable level of the transgene transcript was observed while at high concentrations of blasticidin (10 µg/ml), expression levels increased by ∼10–15 fold (Figure 4B). If the expression of the PfSRIR has a dominant-negative effect, this should be most easily detected at high levels of expression since it is predicted to work in a competitive fashion. We also over-expressed Firefly luciferase as a negative control. Parasites over-expressing the PfSRIR construct were viable and displayed no obvious growth defect. To assay for effects on *var* gene expression, we transfected two parasite lines (subclones of NF54 called A3 and C3) that were previously shown to exhibit relatively stable *var* gene expression patterns [28]. These lines were first transfected and grown under 2 µg/ml blasticidin pressure, after which parasites were synchronized and RNA extracted from ring stages. *var* gene expression was determined by quantitative realtime RT-PCR using a primer set designed to specifically detect each individual *var* gene in the parasite's genome [28], [31]. As expected, over-expression of the luciferase control (by increasing the concentration of blasticidin in the culture media from 2 to 10 µg/ml) did not lead to any pronounced change in *var* gene expression, and the dominantly expressed *var* gene remained dominant in parasites grown at each drug concentration (Figure 4C). In contrast, when parasites were forced to over-express the PfSRIR dominant-negative construct, profound changes in *var* gene expression were observed, specifically leading to *var* gene expression switching in both transfected lines (Figure 4D). This phenotype was observed in multiple independent repeats of the experiment (Figures S3, S4, S5, S6). The fact that no changes were observed when luciferase was over-expressed at similarly high levels indicates that the effect was not induced by selection with blasticidin alone.

Interestingly, in both transfected lines, a single *var* gene (PFL0030c) was strongly upregulated in response to over-expression of the dominant-negative construct. In A3, this gene became expressed at a level equal to the previously dominant transcript (MAL6P1.252) while in C3 it almost entirely replaced the previously active gene (PFD0625c). This *var* gene (PFL0030c or PFL3D7_1200600), also referred to as *var2csa*, is an unusual member of the *var* gene family that is under the control of a unique promoter type (called upsE). In addition, its transcript appears to be under translational control through the presence of an upstream open reading frame within the 5′ leader of the transcript [51], [52]. It is possible that over-expression of the PfSET2 dominant-negative construct specifically induces expression of *var2csa*, or alternatively that this gene simply happens by chance to be preferentially activated in these closely related parasite subclones. A comprehensive set of experiments utilizing the generation of numerous additional transfected parasite lines derived from several distinct geographical isolates will be required to definitively address this question.

## Discussion

The work presented here and in the recent paper by Jiang *et al.* both support a key role for PfSET2 in *var* gene regulation and antigenic variation by *P. falciparum*, however the details of how this histone mark influences *var* gene expression remain to be illuminated. In their analysis of the PfSET2 knockout lines, Jiang and colleagues observed that H3K36me3 is greatly reduced or absent at the mRNA transcription start site of the active *var* gene while this mark covers the promoter regions of silent *var* genes [22]. This led them to propose the simple hypothesis that H3K36me3 is a silencing mark required to keep all but a single member of the *var* gene family in a transcriptional repressed state. However this hypothesis does not offer an explanation for the additional observation that H3K36me3 is heavily present throughout the coding regions of all *var* genes, regardless of their transcriptional state. This differs from other histone marks associated with *var* genes (for example H3K9me3 or H3K9ac) which seem to more strictly mark the promoter and 5′ upstream regions of either active or silent genes. The fact that all *var* genes, both active and silent, are marked by H3K36me3 suggests that this histone modification might have an additional or alternative function than simply being a mark for promoter silencing. One possibility is that H3K36me3 contributes to the *recognition* of individual *var* genes as members of the *var* gene family and therefore subject to mutually exclusive expression. It has previously been observed that when *var* upstream promoters are isolated away from the influence of an adjacent *var* intron, for example when isolated on episomes, they are no longer subject to mutually exclusive expression and become constitutively active [53]–[55]. This implies that they are no longer recognized as members of the *var* gene family. The regulatory activity of *var* introns has been shown to be dependent on their own promoter activity, a property responsible for the production of previously identified noncoding, or “sterile” RNAs [44], [47]. The regulatory properties of *var* introns is disrupted if the sequence responsible for their promoter activity is deleted [56], [57], however proper regulation is restored if the *var* intron is replaced by an alternative promoter sequence [54]. These observations suggest a model in which *var* introns exert their influence not through their specific DNA sequence per se, but rather through the recruitment of RNA pol II, which in turn recruits PfSET2 to the *var* locus, thereby resulting in the deposition of H3K36me3. Thus this model provides a possible mechanistic explanation for several previous observations regarding the role of *var* introns in coordinating expression of the *var* gene family.

The recruitment of PfSET2 during the transcription of noncoding RNAs rather than mRNAs could also provide an explanation for the unusual observation that PfSET2 binds to the CTD when it is unphosphorylated. In model organisms, when transcribing mRNA the CTD undergoes sequential phosphorylation at the S2 and S5 positions of the heptad repeats. While phosphorylation of S5 is necessary for initiation of transcription, it is phosphorylation at S2 that is important for choosing the proper site of termination as well as recruiting factors required for polyadenylation [58]. Interestingly, *var* ncRNAs terminate at a different position than *var* mRNAs transcribed from the same DNA template, and they do not get polyadenylated [27]. This supports the notion that *var* ncRNAs are likely transcribed by an RNA pol II complex in which the CTD is not typically phosphorylated. When these observations are taken together an attractive model emerges for how PfSET2 gets recruited only to specific regions of the genome. Only when transcribing a ncRNA does the CTD assume an atypically or unphosphorylated conformation. The recruitment of PfSET2 and the deposition of the H3K36me3 mark would thus be limited to these regions of the genome. Consistent with this hypothesis, recent genome-wide mapping of H3K36me3 also found this mark at telomeres [22], a region of the genome that has been shown to transcribe ncRNAs in a fashion similar to *var* genes [59], [60]. H3K36me3 was also detected at some members of other multi-copy gene families thought to be involved in antigenic variation, however it is not known if ncRNAs are associated with these regions of the genome. Final validation of this model will require the development of reagents able to detect and differentiate between the different phosphorylated forms of RNA pol II.

The role of RNA pol II in recruiting histone modifiers has become evident through studies of model eukaryotes and likely plays multiple roles in regulating *var* gene expression. Each *var* gene contains three RNA pol II promoters, one upstream of the coding region that is responsible for mRNA production and two located within the intron that result in production of both sense and anti-sense ncRNAs (Figure 3E). The upstream promoter is subject to mutually exclusive expression and thus only recruits RNA pol II when the gene is in the active state and is producing mRNA. However, previous work showed that active transcription from this promoter is necessary for maintenance of epigenetic memory [55], suggesting the possibility that in addition to producing mRNA, the RNA pol II complex also plays a role in the deposition of histone modifications when engaged at this promoter. The intronic promoter that drives expression of anti-sense transcripts similarly only recruits RNA pol II to the single, actively expressed gene, and it does so in ring stage parasites, at the same time the mRNA is transcribed. This promoter activity was described extensively in the recent paper by Jiang *et al.* [22], however how it influences *var* gene expression is not known. The third promoter is responsible for expression of the sense ncRNAs (originally called sterile *RNAs*) that initiate from the introns of *var* genes. These promoters recruit RNA pol II during the late trophozoite and schizont stages and appear to be active at all *var* genes simultaneously [47], [61]. This promoter is the most likely to be responsible for recruiting PfSET2 to *var* loci, given that all *var* loci are marked by H3K36me3 regardless of whether the other two promoters are active or silent. Previous studies have proposed a “pairing” mechanism by which *var* introns and upstream promoters directly interact in a one-to-one basis [53], [62], and DNA elements responsible for pairing were recently identified [63]. Such interactions are predicted to result in the formation of a loop structure that would bend the intron back into close proximity to exon 1, thereby enabling the RNA pol II/PfSET2 complex to deposit the H3K36me3 mark throughout both exons of the gene.

Over-expression of the PfSET2 dominant-negative construct led to accelerated *var* gene expression switching in our transfected lines. The construct was not designed to reduce the enzymatic activity of the endogenous protein, but rather to interfere with its recruitment to the RNA pol II complex, thereby down-regulating its ability to modify chromatin at *var* genes. We hypothesize that H3K36me3 is involved in recognition of *var* genes for inclusion in the mutually exclusive expression pathway. If correct, down-regulation of PfSET2 could “loosen” this control, thereby resulting in accelerated switching. This model also predicts that a full knock-out of PfSET2 would completely disrupt mutually exclusive expression, leading to expression of many or all *var* genes simultaneously. This is exactly what was observed in a recent study describing parasites in which PfSET2 was knocked out [22]. The fact that *var2csa* was preferentially activated in response to over-expression of the PfSET2 dominant-negative construct raises the question of whether this *var* gene occupies a unique position within the regulatory pathway that coordinates *var* gene expression. In addition to being regulated at the translational level and having an upstream promoter region unlike other *var* genes, this gene has also been reported to display an exceptionally high “on” switching rate in certain subclones [64]. These authors proposed that high spontaneous switching rates to this gene combined with translational repression might constitute one component of the *var* gene regulatory pathway. Our data might provide additional evidence of a unique role for *var2csa* in controlling *var* gene expression and antigenic variation.

The lack of SET2 in the genomes of rodent parasites is perhaps more puzzling, although it is consistent with a possible role for PfSET2 in antigenic variation, a process that has been shown to be regulated somewhat differently in parasites of rodents [24]. In our *in vitro* binding assays, PfSET2 binds equally well to both the expanded CTD of *P. falciparum* as well as the much shorter CTD of *P berghei*, conceivably arguing that the CTD expansion in primate parasites is unrelated to interactions with PfSET2. Alternatively, the CTD is thought to interact with several proteins simultaneously during transcription, and thus an extended length could provide it with a greater capacity to recruit additional proteins to the transcriptional complex. Hence the expanded CTD could provide the transcriptional complex the added capacity to recruit PfSET2 in addition to other proteins required for its functionality. This property would not be evident in our *in vitro* binding assays, which utilized individual proteins. A similar interpretation can be made for the yeast system, in which a minimum of eight heptads are required for viability of the organisms, but binding of SET2 is evident with only three in an *in vitro* assay [17]. It is also worth noting that loss of the SET2 ortholog does not necessarily imply that rodent parasites do not possess H3K36me3 in their chromatin, but rather than this particular methyltransferase that binds directly to the unphosphorylated CTD of RNA pol II has been lost. Jiang *et al.* found that knocking out *PfSet2* does not result in a complete loss of H3K36me3 in *P. falciparum* [22], suggesting that an alternative enzyme exists with this methyltransferase activity.

In conclusion, here we provide data to support the role of the histone methyltransferase PfSET2 in regulating *var* gene expression and antigenic variation in *P. falciparum*. Perhaps more importantly, we also propose a mechanism for its recruitment to *var* loci through interactions with RNA pol II, potentially during the production of ncRNAs. These observations provide an additional degree of understanding for how proper chromatin structure is assembled at various genomic positions, and may provide a new paradigm for understanding how other aspects of parasite biology are controlled.

## Supporting Information

Figure S1 Immunofluorescence assay (IFA) of rings and schizonts using anti-H3K36me3 antibodies. Localization of the antibody is shown in red, the parasite nuclei are stained with Dapi (blue). In both stages the antibody detects a single spot in which this histone modification appears to be localized. Similar results were recently reported by Jiang et al (2013). These results contrast with ChIP data that detect H3K36me3 at all *var* loci and telomeres, which have previously been shown to localize at multiple locations within the nucleus. A possible explanation is that in fixed cells, the H3K36me3 epitope might only be accessible to the antibody when it is in open chromatin, and therefore the IFAs only allow visualization of the single active *var* gene. Additional work will be required to determine the cause of this pattern.
(PDF)

Figure S2 The amino acid sequence of the R2 and R3 regions of the C-terminal domain of Rpb1 from *P. falciparum* 3D7. The heptad repeats typical of Rpb1 are underlined, and the serine residues that are sites for phosphorylation are marked with asterisks.
(PDF)

Figure S3 *var* gene family transcription profile from A3 cultures (Figure 4) shown as bar graphs. We used the *var* primer set to detect transcription as designed by Salanti *et al*. *var* gene expression patterns when overexpressing Firefly Luciferase at 2 µg/ml (A) and 10 µg/ml blasticidin (B). *var* gene expression profiles in the presence of the dominant-negative, PfSRI at 2 µg/ml (C) and 10 µg/ml blasticidin (D). The p10 primer pair detects PFL0030c, or *var2csa*, at higher concentrations of blasticidin.
(PDF)

Figure S4 *var* gene family transcription profile for C3 cultures from Figure 4 also shown as bar graphs. The dominant *var* gene in the cultures expressing Luciferase does not change at both 2 µg/ml and 10 µg/ml blasticidin (A and B respectively). Like the A3 experiments, *var2csa* also becomes the dominant expressing *var* gene in the presence of PfSRIR but only at 10 µg/ml blasticidin (C and D).
(PDF)

Figure S5 Transcriptional profile of the *var* gene family after two (A and B) independent increases from 2 µg/ml to 10 µg/ml blasticidin in A3 cultures overexpressing the dominant-negative, PfSRIR. The results in both experiments are similar to those shown in Figure 4D (left 2 pie charts).
(PDF)

Figure S6 An independent transfection of C3 cultures with the constructs expressing Luciferase (A) and the dominant-negative, PfSRIR (B) shows a similar change in *var* gene transcription profile at 10 µg/ml blasticidin.
(PDF)

Figure S7 Chromatin immunoprecipitation data from Figure 3D shown as % input without normalization. Lanes 1 and 2 represent control genes encoding seryl t-RNA synthetase and actin, respectively. Lane 3 represents the gene for circumsporozoite protein. Lane 4 represent CTRP, the gene used for normalization in Figure 3D. The remaining lanes represent regions within the coding portion of both exons of the *var* gene PF3D7_0421100. Black bars show results from chromatin extracted from the C3 line of NF54, in which PF3D7_0421100 is the actively expressed *var* gene. The gray bars show chromatin extracted from the A3 line of NF54 in which this *var* gene is transcriptionally silent. The bars display the mean +/− standard deviation of relative amounts of bound DNA (see methods) from four independent experiments.
(PDF)

Table S1 Primers used for PCR amplification of different regions of PfSET2.
(DOCX)

Table S2 Primers used for Q-PCR amplification to determine expression levels of PfSET2 and the PfSRIR.
(DOC)

## References

[1] Jiang L, Lopez-Barragan MJ, Jiang H, Mu J, Gaur D, et al (2010) Epigenetic control of the variable expression of a Plasmodium falciparum receptor protein for erythrocyte invasion. Proc Natl Acad Sci U S A 107: 2224–2229. 20080673 10.1073/pnas.0913396107 PMC2836689

[2] Cortes A, Carret C, Kaneko O, Yim Lim BY, Ivens A, et al (2007) Epigenetic silencing of Plasmodium falciparum genes linked to erythrocyte invasion. PLoS Pathog 3: e107. 17676953 10.1371/journal.ppat.0030107 PMC1937010

[3] Crowley VM, Rovira-Graells N, Ribas dP, Cortes A (2011) Heterochromatin formation in bistable chromatin domains controls the epigenetic repression of clonally variant Plasmodium falciparum genes linked to erythrocyte invasion. Mol Microbiol 80: 391–406. 21306446 10.1111/j.1365-2958.2011.07574.x

[4] Comeaux CA, Coleman BI, Bei AK, Whitehurst N, Duraisingh MT (2011) Functional analysis of epigenetic regulation of tandem RhopH1/clag genes reveals a role in Plasmodium falciparum growth. Mol Microbiol 80: 378–390. 21320181 10.1111/j.1365-2958.2011.07572.x PMC3622951

[5] Chookajorn T, Dzikowski R, Frank M, Li F, Jiwani AZ, et al (2007) Epigenetic memory at malaria virulence genes. Proc Natl Acad Sci U S A 104: 899–902. 17209011 10.1073/pnas.0609084103 PMC1764221

[6] Lopez-Rubio JJ, Mancio-Silva L, Scherf A (2009) Genome-wide analysis of heterochromatin associates clonally variant gene regulation with perinuclear repressive centers in malaria parasites. Cell Host Microbe 5: 179–190. 19218088 10.1016/j.chom.2008.12.012

[7] Lopez-Rubio JJ, Riviere L, Scherf A (2007) Shared epigenetic mechanisms control virulence factors in protozoan parasites. Curr Opin Microbiol 10: 560–568. 18024150 10.1016/j.mib.2007.10.003

[8] Flueck C, Bartfai R, Volz J, Niederwieser I, Salcedo-Amaya AM, et al (2009) Plasmodium falciparum heterochromatin protein 1 marks genomic loci linked to phenotypic variation of exported virulence factors. PLoS Pathog 5: e1000569. 19730695 10.1371/journal.ppat.1000569 PMC2731224

[9] Cui L, Fan Q, Cui L, Miao J (2008) Histone lysine methyltransferases and demethylases in Plasmodium falciparum. Int J Parasitol 38: 1083–1097. 18299133 10.1016/j.ijpara.2008.01.002 PMC4566933

[10] Tonkin CJ, Carret CK, Duraisingh MT, Voss TS, Ralph SA, et al (2009) Sir2 paralogues cooperate to regulate virulence genes and antigenic variation in Plasmodium falciparum. PLoS Biol 7: e84. 19402747 10.1371/journal.pbio.1000084 PMC2672602

[11] Duraisingh MT, Voss TS, Marty AJ, Duffy MF, Good RT, et al (2005) Heterochromatin silencing and locus repositioning linked to regulation of virulence genes in Plasmodium faiciparum. Cell 121: 13–24. 15820675 10.1016/j.cell.2005.01.036

[12] Suganuma T, Workman JL (2011) Signals and combinatorial functions of histone modifications. Annu Rev Biochem 80: 473–499. 21529160 10.1146/annurev-biochem-061809-175347

[13] Suganuma T, Workman JL (2008) Crosstalk among Histone Modifications. Cell 135: 604–607. 19013272 10.1016/j.cell.2008.10.036

[14] Wagner EJ, Carpenter PB (2012) Understanding the language of Lys36 methylation at histone H3. Nat Rev Mol Cell Biol 13: 115–126. 22266761 10.1038/nrm3274 PMC3969746

[15] Klose RJ, Kallin EM, Zhang Y (2006) JmjC-domain-containing proteins and histone demethylation. Nat Rev Genet 7: 715–727. 16983801 10.1038/nrg1945

[16] Sun XJ, Wei J, Wu XY, Hu M, Wang L, et al (2005) Identification and characterization of a novel human histone H3 lysine 36-specific methyltransferase. J Biol Chem 280: 35261–35271. 16118227 10.1074/jbc.M504012200

[17] Kizer KO, Phatnani HP, Shibata Y, Hall H, Greenleaf AL, et al (2005) A novel domain in Set2 mediates RNA polymerase II interaction and couples histone H3 K36 methylation with transcript elongation. Mol Cell Biol 25: 3305–3316. 15798214 10.1128/MCB.25.8.3305-3316.2005 PMC1069628

[18] Li B, Howe L, Anderson S, Yates JR III, Workman JL (2003) The Set2 histone methyltransferase functions through the phosphorylated carboxyl-terminal domain of RNA polymerase II. J Biol Chem 278: 8897–8903. 12511561 10.1074/jbc.M212134200

[19] Xiao T, Hall H, Kizer KO, Shibata Y, Hall MC, et al (2003) Phosphorylation of RNA polymerase II CTD regulates H3 methylation in yeast. Genes Dev 17: 654–663. 12629047 10.1101/gad.1055503 PMC196010

[20] Cheung V, Chua G, Batada NN, Landry CR, Michnick SW, et al (2008) Chromatin- and transcription-related factors repress transcription from within coding regions throughout the Saccharomyces cerevisiae genome. PLoS Biol 6: e277. 18998772 10.1371/journal.pbio.0060277 PMC2581627

[21] Li B, Jackson J, Simon MD, Fleharty B, Gogol M, et al (2009) Histone H3 lysine 36 dimethylation (H3K36me2) is sufficient to recruit the Rpd3s histone deacetylase complex and to repress spurious transcription. J Biol Chem 284: 7970–7976. 19155214 10.1074/jbc.M808220200 PMC2658090

[22] Jiang L, Mu J, Zhang Q, Ni T, Srinivasan P, et al (2013) PfSETvs methylation of histone H3K36 represses virulence genes in Plasmodium falciparum. Nature 499: 223–227. 23823717 10.1038/nature12361 PMC3770130

[23] Kishore SP, Perkins SL, Templeton TJ, Deitsch KW (2009) An unusual recent expansion of the C-terminal domain of RNA polymerase II in primate malaria parasites features a motif otherwise found only in mammalian polymerases. J Mol Evol 68: 706–714. 19449052 10.1007/s00239-009-9245-2 PMC3622039

[24] Cunningham D, Fonager J, Jarra W, Carret C, Preiser P, et al (2009) Rapid changes in transcription profiles of the Plasmodium yoelii yir multigene family in clonal populations: lack of epigenetic memory? PLoS ONE 4: e4285. 19173007 10.1371/journal.pone.0004285 PMC2628738

[25] Scherf A, Lopez-Rubio JJ, Riviere L (2008) Antigenic variation in Plasmodium falciparum. Annu Rev Microbiol 62: 445–470. 18785843 10.1146/annurev.micro.61.080706.093134

[26] Shechter D, Dormann HL, Allis CD, Hake SB (2007) Extraction, purification and analysis of histones. Nat Protoc 2: 1445–1457. 17545981 10.1038/nprot.2007.202

[27] Epp C, Li F, Howitt CA, Chookajorn T, Deitsch KW (2009) Chromatin associated sense and antisense noncoding RNAs are transcribed from the var gene family of virulence genes of the malaria parasite Plasmodium falciparum. RNA 15: 116–127. 19037012 10.1261/rna.1080109 PMC2612763

[28] Frank M, Dzikowski R, Amulic B, Deitsch K (2007) Variable switching rates of malaria virulence genes are associated with chromosomal position. Mol Microbiol 64: 1486–1498. 17555435 10.1111/j.1365-2958.2007.05736.x PMC3634120

[29] Dzikowski R, Frank M, Deitsch K (2006) Mutually Exclusive Expression of Virulence Genes by Malaria Parasites Is Regulated Independently of Antigen Production. PLoS Pathog 2: e22. 16518466 10.1371/journal.ppat.0020022 PMC1386720

[30] Nkrumah LJ, Muhle RA, Moura PA, Ghosh P, Hatfull GF, et al (2006) Efficient site-specific integration in Plasmodium falciparum chromosomes mediated by mycobacteriophage Bxb1 integrase. Nat Methods 3: 615–621. 16862136 10.1038/nmeth904 PMC2943413

[31] Salanti A, Staalsoe T, Lavstsen T, Jensen ATR, Sowa MPK, et al (2003) Selective upregulation of a single distinctly structured var gene in chondroitin sulphate A-adhering Plasmodium falciparum involved in pregnancy-associated malaria. Molecular Microbiology 49: 179–191. 12823820 10.1046/j.1365-2958.2003.03570.x

[32] Dzikowski R, Templeton TJ, Deitsch K (2006) Variant antigen gene expression in malaria. Cell Microbiol 8: 1371–1381. 16848786 10.1111/j.1462-5822.2006.00760.x

[33] Hampsey M, Reinberg D (2003) Tails of intrigue: phosphorylation of RNA polymerase II mediates histone methylation. Cell 113: 429–432. 12757703 10.1016/s0092-8674(03)00360-x

[34] Bischoff E, Vaquero C (2010) In silico and biological survey of transcription-associated proteins implicated in the transcriptional machinery during the erythrocytic development of Plasmodium falciparum. BMC Genomics 11: 34. 20078850 10.1186/1471-2164-11-34 PMC2821373

[35] Issar N, Ralph SA, Mancio-Silva L, Keeling C, Scherf A (2009) Differential sub-nuclear localisation of repressive and activating histone methyl modifications in P. falciparum. Microbes Infect 11: 403–407. 19136073 10.1016/j.micinf.2008.12.010

[36] Trelle MB, Salcedo-Amaya AM, Cohen AM, Stunnenberg HG, Jensen ON (2009) Global histone analysis by mass spectrometry reveals a high content of acetylated lysine residues in the malaria parasite Plasmodium falciparum. J Proteome Res 8: 3439–3450. 19351122 10.1021/pr9000898

[37] Cui L, Miao J (2010) Chromatin-mediated epigenetic regulation in the malaria parasite Plasmodium falciparum. Eukaryot Cell 9: 1138–1149. 20453074 10.1128/EC.00036-10 PMC2918932

[38] Kishore SP, Stiller JW, Deitsch KW (2013) Horizontal gene transfer of epigenetic machinery and evolution of parasitism in the malaria parasite Plasmodium falciparum and other apicomplexans. BMC Evol Biol 13: 37. 23398820 10.1186/1471-2148-13-37 PMC3598677

[39] Vojnic E, Simon B, Strahl BD, Sattler M, Cramer P (2006) Structure and carboxyl-terminal domain (CTD) binding of the Set2 SRI domain that couples histone H3 Lys36 methylation to transcription. J Biol Chem 281: 13–15. 16286474 10.1074/jbc.C500423200

[40] Chapman RD, Heidemann M, Hintermair C, Eick D (2008) Molecular evolution of the RNA polymerase II CTD. Trends Genet 24: 289–296. 18472177 10.1016/j.tig.2008.03.010

[41] Egloff S, Dienstbier M, Murphy S (2012) Updating the RNA polymerase CTD code: adding gene-specific layers. Trends Genet 28: 333–341. 22622228 10.1016/j.tig.2012.03.007

[42] Le Roch KG, Zhou YY, Blair PL, Grainger M, Moch JK, et al (2003) Discovery of gene function by expression profiling of the malaria parasite life cycle. Science 301: 1503–1508. 12893887 10.1126/science.1087025

[43] Bozdech Z, Llinas M, Pulliam BL, Wong ED, Zhu J, et al (2003) The transcriptome of the intraerythrocytic developmental cycle of Plasmodium falciparum. PLoS Biol 1: E5. 12929205 10.1371/journal.pbio.0000005 PMC176545

[44] Kyes S, Christodoulou Z, Pinches R, Kriek N, Horrocks P, et al (2007) Plasmodium falciparum var gene expression is developmentally controlled at the level of RNA polymerase II-mediated transcription initiation. Mol Microbiol 63: 1237–1247. 17257309 10.1111/j.1365-2958.2007.05587.x

[45] Baruch DI, Pasloske BL, Singh HB, Bi X, Ma XC, et al (1995) Cloning the *P. falciparum* gene encoding PfEMP1, a malarial variant antigen and adherence receptor on the surface of parasitized human erythrocytes. Cell 82: 77–87. 7541722 10.1016/0092-8674(95)90054-3

[46] Smith JD, Chitnis CE, Craig AG, Roberts DJ, Hudson-Taylor DE, et al (1995) Switches in expression of *Plasmodium falciparum var* genes correlate with changes in antigenic and cytoadherent phenotypes of infected erythrocytes. Cell 82: 101–110. 7606775 10.1016/0092-8674(95)90056-x PMC3730239

[47] Su X, Heatwole VM, Wertheimer SP, Guinet F, Herrfeldt JV, et al (1995) A large and diverse gene family (*var*) encodes 200–350 kD proteins implicated in the antigenic variation and cytoadherence of *Plasmodium falciparum*-infected erythrocytes. Cell 82: 89–100. 7606788 10.1016/0092-8674(95)90055-1

[48] Enderes C, Kombila D, Dal Bianco M, Dzikowski R, Kremsner P, et al (2011) Var Gene promoter activation in clonal Plasmodium falciparum isolates follows a hierarchy and suggests a conserved switching program that is independent of genetic background. J Infect Dis 204: 1620–1631. 21926380 10.1093/infdis/jir594

[49] Fastman Y, Noble R, Recker M, Dzikowski R (2012) Erasing the epigenetic memory and beginning to switch–the onset of antigenic switching of var genes in Plasmodium falciparum. PLoS ONE 7: e34168. 22461905 10.1371/journal.pone.0034168 PMC3312910

[50] Epp C, Raskolnikov D, Deitsch KW (2008) A regulatable transgene expression system for cultured Plasmodium falciparum parasites. Malar J 7: 86. 18492282 10.1186/1475-2875-7-86 PMC2409362

[51] Amulic B, Salanti A, Lavstsen T, Nielsen MA, Deitsch KW (2009) An upstream open reading frame controls translation of var2csa, a gene implicated in placental malaria. PLoS Pathog 5: e1000256. 19119419 10.1371/journal.ppat.1000256 PMC2603286

[52] Bancells C, Deitsch KW (2013) A molecular switch in the efficiency of translation reinitiation controls expression of var2csa, a gene implicated in pregnancy associated malaria. Mol Microbiol [epub ahead of print]. 10.1111/mmi.12379 PMC3938558 23980802

[53] Frank M, Dzikowski R, Constantini D, Amulic B, Burdougo E, et al (2006) Strict pairing of var promoters and introns is required for var gene silencing in the malaria parasite plasmodium falciparum. J Biol Chem 281: 9942–9952. 16455655 10.1074/jbc.M513067200 PMC3941977

[54] Dzikowski R, Li F, Amulic B, Eisberg A, Frank M, et al (2007) Mechanisms underlying mutually exclusive expression of virulence genes by malaria parasites. EMBO Rep 8: 959–965. 17762879 10.1038/sj.embor.7401063 PMC2002552

[55] Dzikowski R, Deitsch KW (2008) Active transcription is required for maintenance of epigenetic memory in the malaria parasite Plasmodium falciparum. J Mol Biol 382: 288–297. 18656490 10.1016/j.jmb.2008.07.015 PMC3614407

[56] Calderwood MS, Gannoun-Zaki L, Wellems TE, Deitsch KW (2003) Plasmodium falciparum var genes are regulated by two regions with separate promoters, one upstream of the coding region and a second within the intron. Journal of Biological Chemistry 278: 34125–34132. 12832422 10.1074/jbc.M213065200

[57] Gannoun-Zaki L, Jost A, Mu JB, Deitsch KW, Wellems TE (2005) A silenced Plasmodium falciparum var promoter can be activated in vivo through spontaneous deletion of a silencing element in the intron. Eukaryotic Cell 4: 490–492. 15701812 10.1128/EC.4.2.490-492.2005 PMC549332

[58] Gudipati RK, Villa T, Boulay J, Libri D (2008) Phosphorylation of the RNA polymerase II C-terminal domain dictates transcription termination choice. Nat Struct Mol Biol 15: 786–794. 18660821 10.1038/nsmb.1460

[59] Broadbent KM, Park D, Wolf AR, Van Tyne D, Sims JS, et al (2011) A global transcriptional analysis of Plasmodium falciparum malaria reveals a novel family of telomere-associated lncRNAs. Genome Biol 12: R56. 21689454 10.1186/gb-2011-12-6-r56 PMC3218844

[60] Sierra-Miranda M, Delgadillo DM, Mancio-Silva L, Vargas M, Villegas-Sepulveda N, et al (2012) Two long non-coding RNAs generated from subtelomeric regions accumulate in a novel perinuclear compartment in Plasmodium falciparum. Mol Biochem Parasitol 185: 36–47. 22721695 10.1016/j.molbiopara.2012.06.005 PMC7116675

[61] Kyes SA, Christodoulou Z, Raza A, Horrocks P, Pinches R, et al (2003) A well-conserved Plasmodium falciparum var gene shows an unusual stage-specific transcript pattern. Molecular Microbiology 48: 1339–1348. 12787360 10.1046/j.1365-2958.2003.03505.x PMC2869446

[62] Swamy L, Amulic B, Deitsch KW (2011) Plasmodium falciparum var gene silencing is determined by cis DNA elements that form stable and heritable interactions. Eukaryot Cell 10: 530–539. 21317310 10.1128/EC.00329-10 PMC3127639

[63] Avraham I, Schreier J, Dzikowski R (2012) Insulator-like pairing elements regulate silencing and mutually exclusive expression in the malaria parasite Plasmodium falciparum. Proc Natl Acad Sci U S A 109: E3678–E3686. 23197831 10.1073/pnas.1214572109 PMC3535642

[64] Mok BW, Ribacke U, Rasti N, Kironde F, Chen Q, et al (2008) Default Pathway of var2csa switching and translational repression in Plasmodium falciparum. PLoS ONE 3: e1982. 18431472 10.1371/journal.pone.0001982 PMC2292259

