# Supplementary material for: Recruitment of PfSET2 by RNA Polymerase II to Variant Antigen Encoding Loci Contributes to Antigenic Variation in P. falciparum
Source: PLoS Pathog. 2014 Jan 2;10(1):e1003854. doi: 10.1371/journal.ppat.1003854 (PMC3879369; doi:10.1371/journal.ppat.1003854)
Supplement: Figure S1 — Immunofluorescence assay (IFA) of rings and schizonts using anti-H3K36me3 antibodies. Localization of the antibody is shown in red, the parasite nuclei are stained with Dapi (blue). In both stages the antibody detects a single spot in which this histone modification appears to be localized. Similar results were recently reported by Jiang et al (2013). These results contrast with ChIP data that detect H3K36me3 at all var loci and telomeres, which have previously been shown to localize at multiple locations within the nucleus. A possible explanation is that in fixed cells, the H3K36me3 epitope might only be accessible to the antibody when it is in open chromatin, and therefore the IFAs only allow visualization of the single active var gene. Additional work will be required to determine the cause of this pattern. (PDF) [file ppat.1003854.s001.pdf]

Rings Schizonts

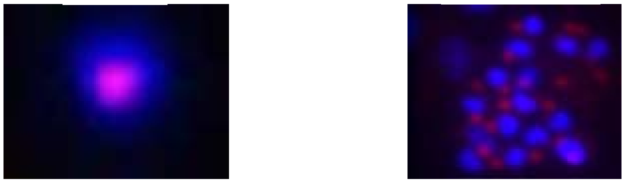

Figure S1. Immunofluorescence assay (IFA) of rings and schizonts using anti-H3K36me3 antibodies. Localization of the antibody is shown in red, the parasite nuclei are stained with Dapi (blue). In both stages the antibody detects a single spot in which this histone modification appears to be localized. Similar results were recently reported by Jiang et al (2013). These results contrast with ChIP data that detect H3K36me3 at all *var* loci and telomeres, which have previously been shown to localize at multiple locations within the nucleus. A possible explanation is that in fixed cells, the H3K36me3 epitope might only be accessible to the antibody when it is in open chromatin, and therefore the IFAs only allow visualization of the single active *var* gene. Additional work will be required to determine the cause of this pattern.

IFA was performed as described by Dahan-Pasternak et al (2013). Briefly, *P. falciparum* cells were washed and fixed with 4% paraformaldehye and 0.075% glutaraldehyde in PBS. Fixed parasites were pelleted, permeabilized with 0.1% Triton X-100 and then blocked with 3% PBS-BSA at 4°C. Cells were then incubated with primary antibody (rabbit anti-H3K36me3, see main text, 1:2000 dilution), washed and incubated with secondary antibody (Alexa-594 conjugated goat anti-rabbit, Invitrogen, 1:500 dilution). Samples were washed, put on coverslips and mounted in VectaShield DAPI stain. Fluorescence images were obtained using an Olympus BX-51 fluorescence microscope at 100X magnification with an Optronics digital imaging system.

Dahan-Pasternak N, Nasereddin A, Kolevzon N, Pe'er M, Wong W, Shinder V, Turnbull L, Whitchurch CB, Elbaum M, Timgilberger W, Yavin E, Baum J,Dzikowski R. (2013) PfSec13 is an unusual chromatin-associated nucleoporin of *Plasmodium falciparum* that is essential for parasite proliferation in human erythrocytes. *J Cell Sci*. **126:** 3055-69.

Jiang L, Mu J, Zhang Q, Ni T, Srinivasan P, Rayavara K, Yang W, Turner L, Lavstsen T, Theander TG, Peng W, Wei G, Jing Q, Wakabayashi Y, Bansal A, Luo Y, Ribeiro JM, Scherf A, Aravind L, Zhu J, Zhao K, Miller LH (2013) PfSETvs methylation of histone H3K36 represses virulence genes in Plasmodium falciparum. *Nature* **499:** 223-227.
