# Supplementary material for: Recruitment of PfSET2 by RNA Polymerase II to Variant Antigen Encoding Loci Contributes to Antigenic Variation in P. falciparum
Source: PLoS Pathog. 2014 Jan 2;10(1):e1003854. doi: 10.1371/journal.ppat.1003854 (PMC3879369; doi:10.1371/journal.ppat.1003854)
Supplement: Figure S2 — The amino acid sequence of the R2 and R3 regions of the C-terminal domain of Rpb1 from P. falciparum 3D7. The heptad repeats typical of Rpb1 are underlined, and the serine residues that are sites for phosphorylation are marked with asterisks. (PDF) [file ppat.1003854.s002.pdf]

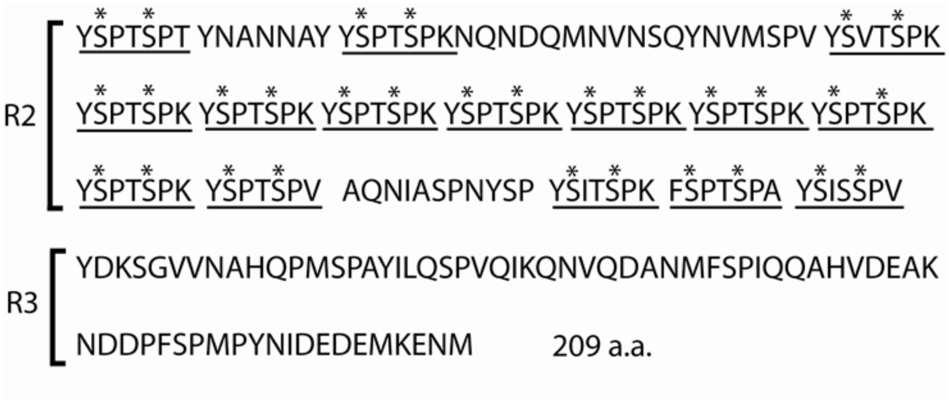

Figure S2. The amino acid sequence of the R2 and R3 regions of the C-terminal domain of Rpb1 from *P. falciparum* 3D7. The heptad repeats typical of Rpb1 are underlined, and the serine residues that are sites for phosphorylation are marked with asterisks.
