# Supplementary material for: Recruitment of PfSET2 by RNA Polymerase II to Variant Antigen Encoding Loci Contributes to Antigenic Variation in P. falciparum
Source: PLoS Pathog. 2014 Jan 2;10(1):e1003854. doi: 10.1371/journal.ppat.1003854 (PMC3879369; doi:10.1371/journal.ppat.1003854)
Supplement: Figure S3 — var gene family transcription profile from A3 cultures (Figure 4) shown as bar graphs. We used the var primer set to detect transcription as designed by Salanti et al. var gene expression patterns when overexpressing Firefly Luciferase at 2 µg/ml (A) and 10 µg/ml blasticidin (B). var gene expression profiles in the presence of the dominant-negative, PfSRI at 2 µg/ml (C) and 10 µg/ml blasticidin (D). The p10 primer pair detects PFL0030c, or var2csa, at higher concentrations of blasticidin. (PDF) [file ppat.1003854.s003.pdf]

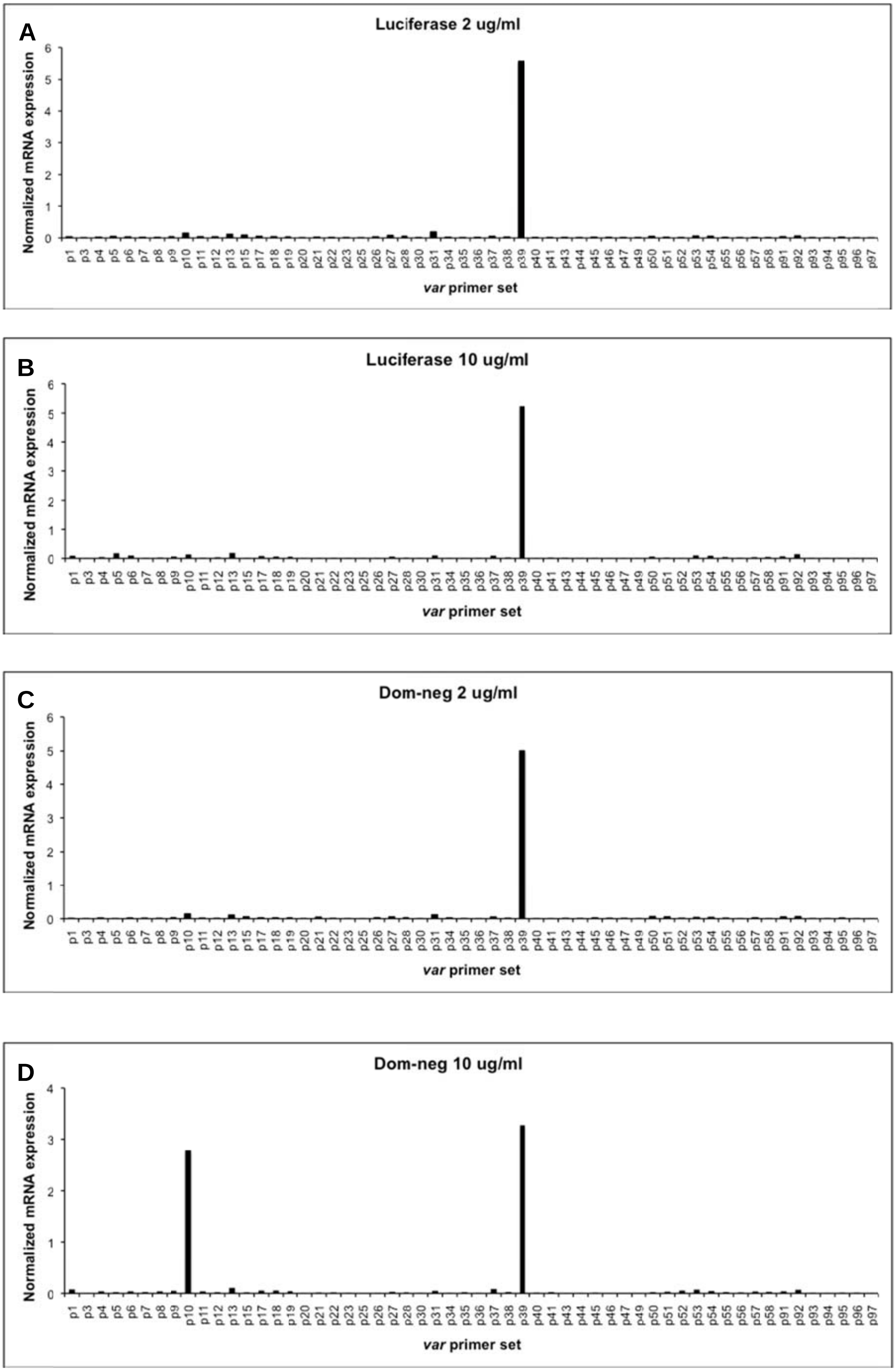
A
Luciferase 2 ug/ml
B
Luciferase 10 ug/ml
C
Dom-neg 2 ug/ml
D
Dom-neg 10 ug/ml
Normalized mRNA expression
var primer set
p1 p3 p4 p5 p6 p7 p8 p9 p10 p11 p12 p13 p15 p17 p18 p19 p20 p21 p22 p23 p25 p26 p27 p28 p30 p31 p34 p35 p36 p37 p38 p39 p40 p41 p43 p44 p45 p46 p47 p49 p50 p51 p52 p53 p54 p55 p56 p57 p58 p91 p92 p93 p94 p95 p96 p97

Figure S3. *var* gene family transcription profile from A3 cultures (Figure 4) shown as bar graphs. We used the *var* primer set to detect transcription as designed by Salanti *et al*. *var* gene expression patterns when overexpressing Firefly Luciferase at 2 μg/ml (A) and 10 μg/ml blasticidin (B). *var* gene expression profiles in the presence of the dominant-negative, PfSRI at 2 μg/ml (C) and 10 μg/ml blasticidin (D). The p10 primer pair detects PFL0030c, or *var2csa,* at higher concentrations of blasticidin.
