# Supplementary material for: Recruitment of PfSET2 by RNA Polymerase II to Variant Antigen Encoding Loci Contributes to Antigenic Variation in P. falciparum
Source: PLoS Pathog. 2014 Jan 2;10(1):e1003854. doi: 10.1371/journal.ppat.1003854 (PMC3879369; doi:10.1371/journal.ppat.1003854)
Supplement: Figure S4 — var gene family transcription profile for C3 cultures from Figure 4 also shown as bar graphs. The dominant var gene in the cultures expressing Luciferase does not change at both 2 µg/ml and 10 µg/ml blasticidin (A and B respectively). Like the A3 experiments, var2csa also becomes the dominant expressing var gene in the presence of PfSRIR but only at 10 µg/ml blasticidin (C and D). (PDF) [file ppat.1003854.s004.pdf]

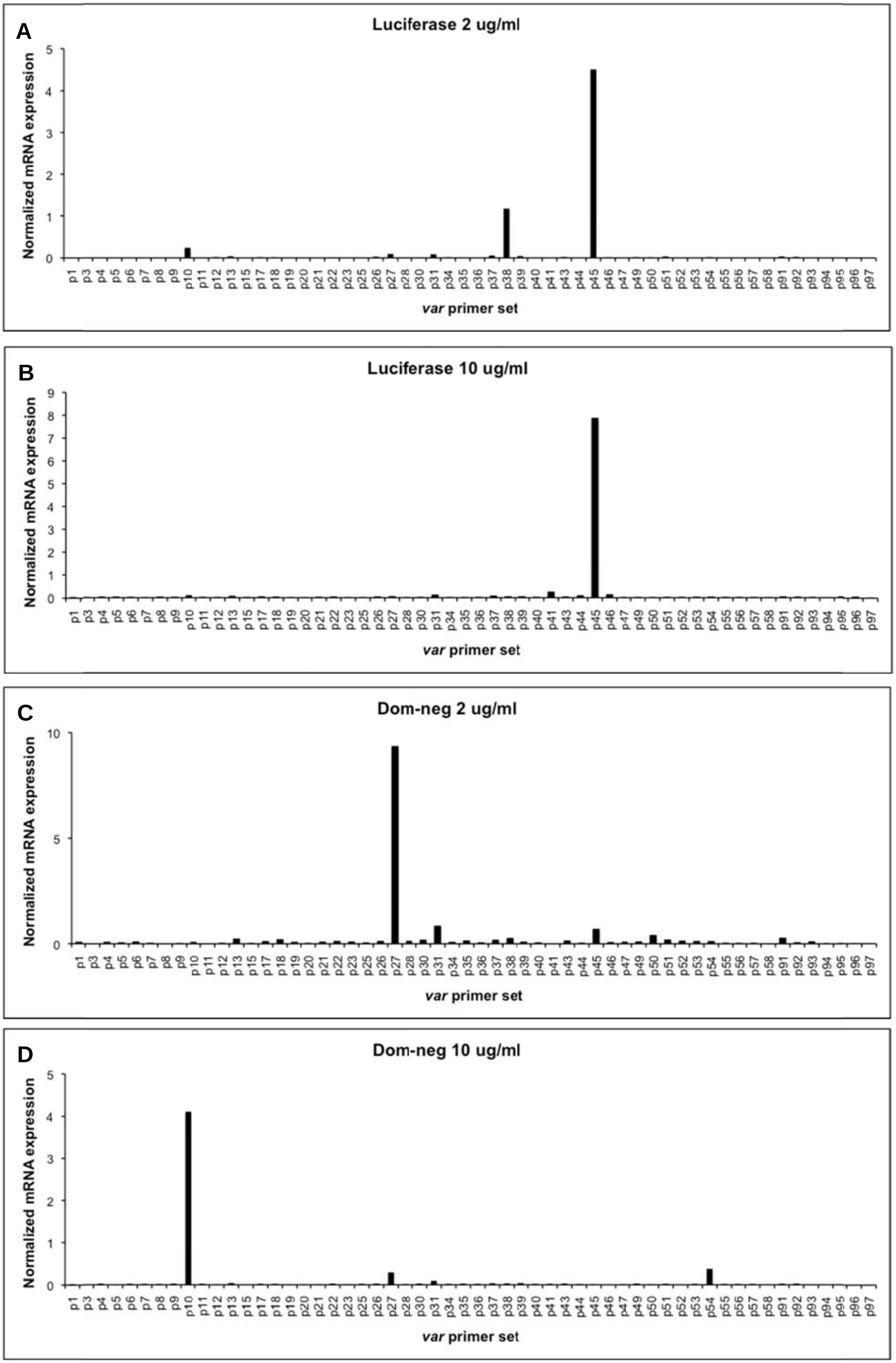
A
Luciferase 2 ug/ml
Normalized mRNA expression
var primer set
B
Luciferase 10 ug/ml
Normalized mRNA expression
var primer set
C
Dom-neg 2 ug/ml
Normalized mRNA expression
var primer set
D
Dom-neg 10 ug/ml
Normalized mRNA expression
var primer set

Figure S4. *var* gene family transcription profile for C3 cultures from Figure 4 also shown as bar graphs. The dominant *var* gene in the cultures expressing Luciferase does not change at both 2 μg/ml and 10μg/ml blasticidin (A and B respectively). Like the A3 experiments, *var2csa* also becomes the dominant expressing *var* gene in the presence of PfSRIR but only at 10μg/ml blasticidin (C and D).
