# Supplementary material for: Recruitment of PfSET2 by RNA Polymerase II to Variant Antigen Encoding Loci Contributes to Antigenic Variation in P. falciparum
Source: PLoS Pathog. 2014 Jan 2;10(1):e1003854. doi: 10.1371/journal.ppat.1003854 (PMC3879369; doi:10.1371/journal.ppat.1003854)
Supplement: Figure S5 — Transcriptional profile of the var gene family after two (A and B) independent increases from 2 µg/ml to 10 µg/ml blasticidin in A3 cultures overexpressing the dominant-negative, PfSRIR. The results in both experiments are similar to those shown in Figure 4D (left 2 pie charts). (PDF) [file ppat.1003854.s005.pdf]

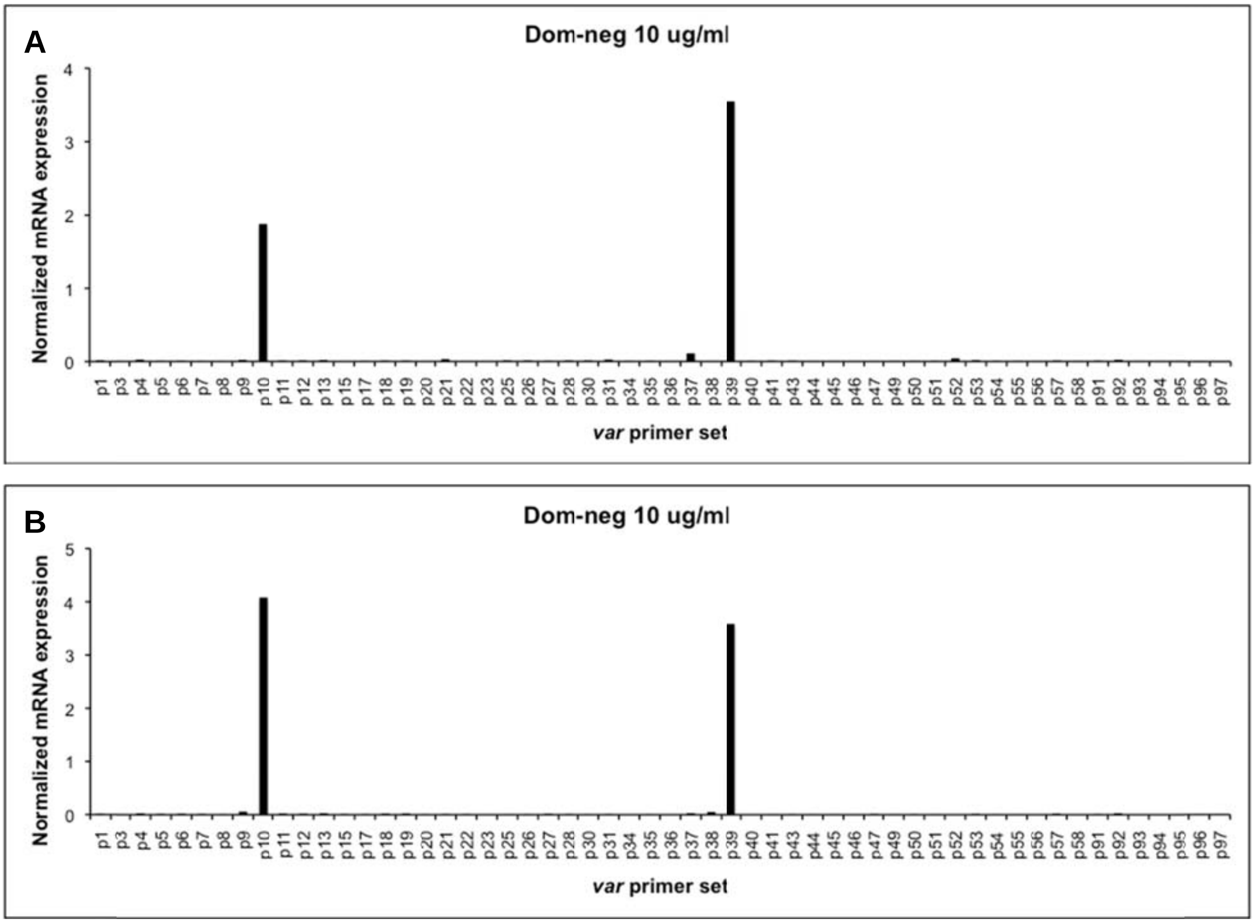

Figure S5. Transcriptional profile of the *var* gene family after two (A and B) independent increases from 2 μg/ml to 10 μg/ml blasticidin in A3 cultures overexpressing the dominant-negative, PfSRIR. The results in both experiments are similar to those shown in Figure 4D (left 2 pie charts).
