# Supplementary material for: Recruitment of PfSET2 by RNA Polymerase II to Variant Antigen Encoding Loci Contributes to Antigenic Variation in P. falciparum
Source: PLoS Pathog. 2014 Jan 2;10(1):e1003854. doi: 10.1371/journal.ppat.1003854 (PMC3879369; doi:10.1371/journal.ppat.1003854)
Supplement: Figure S6 — An independent transfection of C3 cultures with the constructs expressing Luciferase (A) and the dominant-negative, PfSRIR (B) shows a similar change in var gene transcription profile at 10 µg/ml blasticidin. (PDF) [file ppat.1003854.s006.pdf]

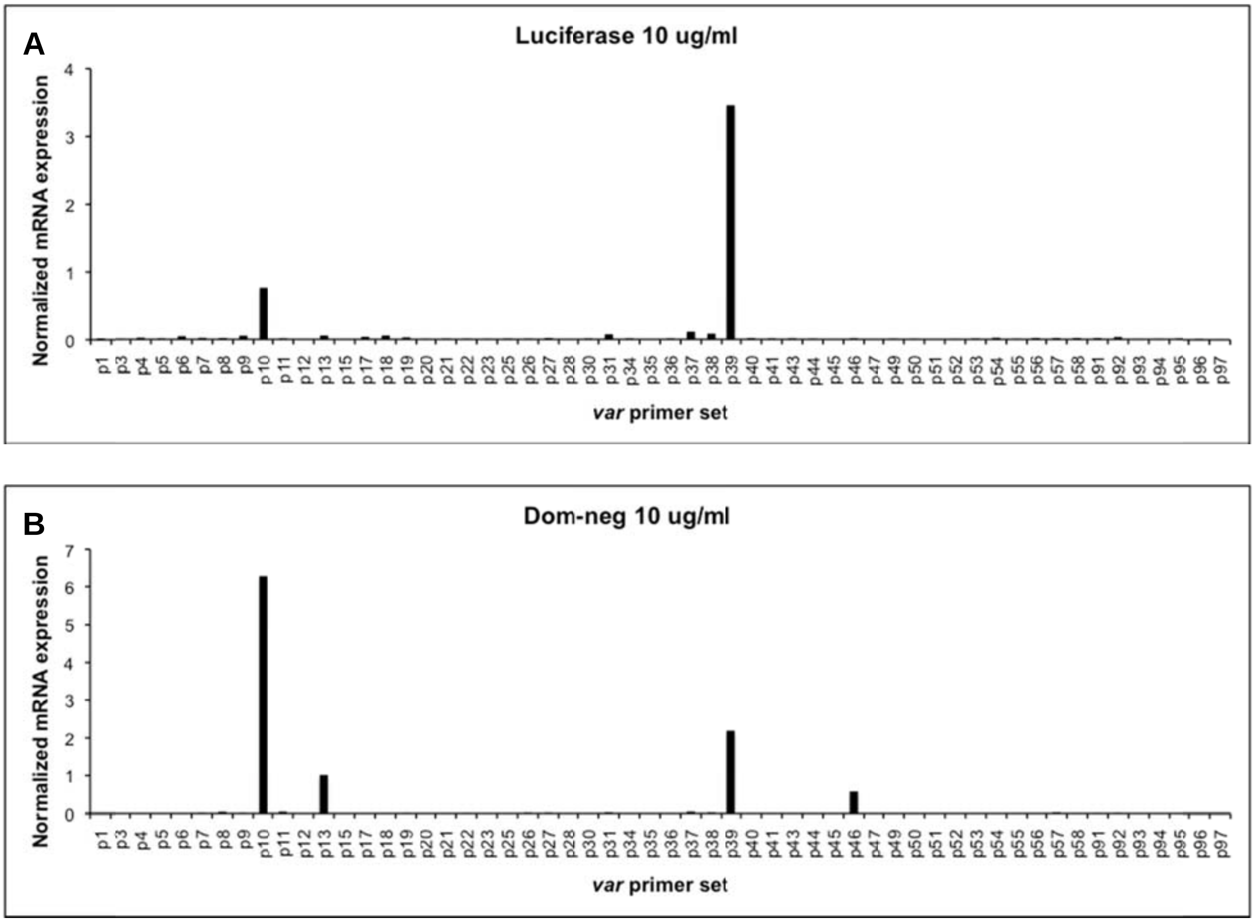

Figure S6. An independent transfection of C3 cultures with the constructs expressing Luciferase (A) and the dominant-negative, PfSRIR (B) shows a similar change in *var* gene transcription profile at 10 μg/ml blasticidin.
