# Supplementary material for: Recruitment of PfSET2 by RNA Polymerase II to Variant Antigen Encoding Loci Contributes to Antigenic Variation in P. falciparum
Source: PLoS Pathog. 2014 Jan 2;10(1):e1003854. doi: 10.1371/journal.ppat.1003854 (PMC3879369; doi:10.1371/journal.ppat.1003854)
Supplement: Figure S7 — Chromatin immunoprecipitation data from Figure 3D shown as % input without normalization. Lanes 1 and 2 represent control genes encoding seryl t-RNA synthetase and actin, respectively. Lane 3 represents the gene for circumsporozoite protein. Lane 4 represent CTRP, the gene used for normalization in Figure 3D. The remaining lanes represent regions within the coding portion of both exons of the var gene PF3D7_0421100. Black bars show results from chromatin extracted from the C3 line of NF54, in which PF3D7_0421100 is the actively expressed var gene. The gray bars show chromatin extracted from the A3 line of NF54 in which this var gene is transcriptionally silent. The bars display the mean +/− standard deviation of relative amounts of bound DNA (see methods) from four independent experiments. (PDF) [file ppat.1003854.s007.pdf]

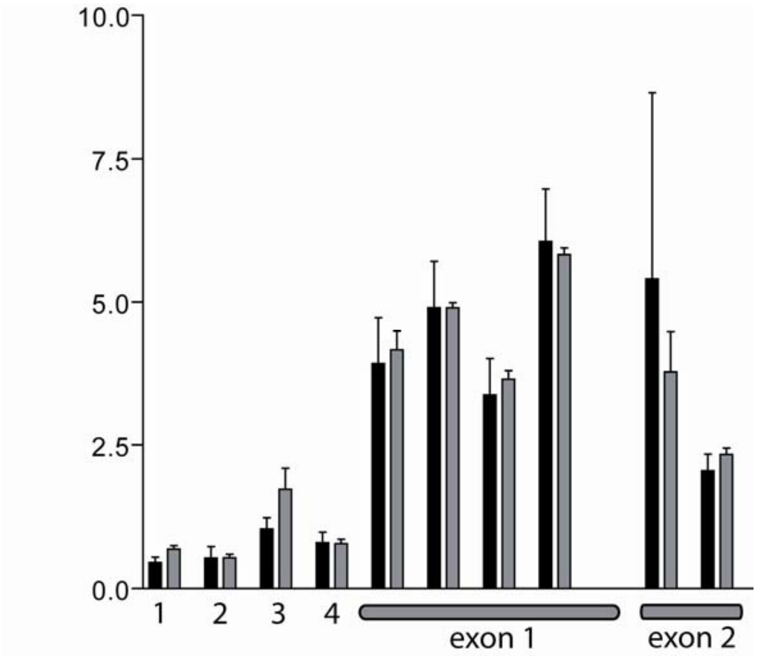

Figure S7. Chromatin immunoprecipitation data from Figure 3D shown as % input without normalization. Lanes 1 and 2 represent control genes encoding seryl t-RNA synthetase and actin, respectively. Lane 3 represents the gene for circumsporozoite protein. Lane 4 represent CTRP, the gene used for normalization in Figure 3D. The remaining lanes represent regions within the coding portion of both exons of the *var* gene PF3D7_0421100. Black bars show results from chromatin extracted from the C3 line of NF54, in which PF3D7_0421100 is the actively expressed *var* gene. The gray bars show chromatin extracted from the A3 line of NF54 in which this *var* gene is transcriptionally silent. The bars display the mean +/- standard deviation of relative amounts of bound DNA (see methods) from four independent experiments.
