# Supplementary material for: Recruitment of PfSET2 by RNA Polymerase II to Variant Antigen Encoding Loci Contributes to Antigenic Variation in P. falciparum
Source: PLoS Pathog. 2014 Jan 2;10(1):e1003854. doi: 10.1371/journal.ppat.1003854 (PMC3879369; doi:10.1371/journal.ppat.1003854)
Supplement: Table S1 — Primers used for PCR amplification of different regions of PfSET2. (DOCX) [file ppat.1003854.s008.docx]

**Supplementary Table S1.** Primers used for PCR amplification of different regions of PfSET2.

| PfSet2 fragments | PfSet2 primers |
| --- | --- |
| Set2 (1-359) | Forward: 5’CGCGGATCCATGGAATACAAACTTTTTAAGAA3’ |
|  | Reverse: 5’ATTTGCGGCCGC**TCA**AGCGTAATCTGGAACATCGTATGGGTATTCACCATCTTGTTCTCTTCGATG3’ |
| Set2 (351-828) | Forward: 5’CGCGGATCCATGCATCGAAGAGAACAAGATGGTG3’ |
|  | Reverse: 5’ATTTGCGGCCGC**TCA**AGCGTAATCTGGAACATCGTATGGGTAACATGCTTTATGAAATGTTCTACC3’ |
| Set2 (1060-1535) | Forward: 5’ CGCGGATCCATGAAAAAGGATCTGCTTCGAGATG3’ |
|  | Reverse: 5’ATTTGCGGCCGC**TCA**AGCGTAATCTGGAACATCGTATGGGTAAAATTGTTCGTCTCCTTTCATAAATTC3’ |
| Set2 (1540-2080) | Forward: 5’ CGCGGATCCATGAATTTAGAAAGTAACGAAAAAAATAC3’ |
|  | Reverse: 5’ATTTGCGGCCGC**TCA**AGCGTAATCTGGAACATCGTATGGGTA CAAATTACATTCCCCTTGACATAAAC3’ |
| Set2 (2264-2548) | Forward: 5’ CGCGGATCCAAAGGAGAAAGTTCAGGAGCTAGC3’ |
|  | Reverse: 5’ATTTGCGGCCGC**TCA**AGCGTAATCTGGAACATCGTATGGGTA TATTTTTACATATTTGTACTGAAATTTCC3’ |
|  |  |
| ySRI (619-734) | Forward: 5’CGCGGATCCATGAAAACGGTCTCCCAGTCCCAAAG3’ |
|  | Reverse: 5’ATTTGCGGCCGC**TCA**AGCGTAATCTGGAACATCGTATGGGTATGATGATGTTGAAGGTGGAGG3’ |

Set 2 (and ySRI) primers

Key:

f = forward

r = reverse

BamHI restriction site = GGATCC

NotI restriction site = GCGGCCGC

HA tag reverse complement (5’ – 3’) = AGCGTAATCTGGAACATCGTATGGGTA

extra nucleotides = CGC and ATTT

start codon = ATG

stop codon = **TCA** (reverse complement of TGA)

Primers read 5’ – 3’
