# Supplementary material for: Recruitment of PfSET2 by RNA Polymerase II to Variant Antigen Encoding Loci Contributes to Antigenic Variation in P. falciparum
Source: PLoS Pathog. 2014 Jan 2;10(1):e1003854. doi: 10.1371/journal.ppat.1003854 (PMC3879369; doi:10.1371/journal.ppat.1003854)
Supplement: Table S2 — Primers used for Q-PCR amplification to determine expression levels of PfSET2 and the PfSRIR. (DOC) [file ppat.1003854.s009.doc]

**Supplementary Table S2.** Primers used for Q-PCR amplification to determine expression levels of PfSET2 and the PfSRIR.

| PfSRIR real-time primers | Forward: GGGAAAGAAACATATCCCCAAG |
| --- | --- |
|  | Reverse: CCCGTGCTTTCATCTTGTCT |
| PfSET2 real-time primers | Forward: TTGAAGATGGAAAACATCATTG |
|  | Reverse: CATATAAGAAGCATCAGGAATGGA |
